# Who are the beneficiaries and what are the reasons for non-utilization of care respite and support services? A cross-sectional study on family caregivers

**DOI:** 10.1186/s12913-021-06651-6

**Published:** 2021-07-02

**Authors:** Jianan Huang, Nadja Münzel, Anke Scheel-Sailer, Armin Gemperli

**Affiliations:** 1 Swiss Paraplegic Research, Guido A. Zäch Strasse 4, 6207 Nottwil, Switzerland; 2 Department of Health Sciences and Medicine, University of Lucerne, Frohburgstrasse 3, 6002 Lucerne, Switzerland; 3 ParaHelp AG, Guido A. Zäch Strasse 1, 6207 Nottwil, Switzerland; 4 Swiss Paraplegic Centre, Guido A. Zäch Strasse 1, 6207 Nottwil, Switzerland; 5 Center of Primary and Community Care, University of Lucerne, Frohburgstrasse 3, 6002 Lucerne, Switzerland

**Keywords:** Family caregivers, Respite care, Chronic conditions, Classification tree

## Abstract

**Background:**

Family caregivers assume substantial caregiving responsibilities for persons with chronic conditions, such as individuals with spinal cord injury, which leads to negative impacts on their lives. Respite care and other services are provided as a temporary relief and support for them. Design of appropriate respite care programs depends on identification of beneficiary subgroups for the different types of service. This study aimed to quantify the uptake of different respite and support services for family caregivers, the reasons for non-use, and to explore the respective predictors.

**Methods:**

A cross-sectional survey of family caregivers of persons with spinal cord injury was conducted nationwide in Switzerland. The use of 11 different respite and support services during the previous 12 months was investigated, along with caregivers’ reasons for not using any respite. Classification trees were used to characterize the beneficiaries and reasons for not using respite.

**Results:**

About a third of family caregivers used at least one type of respite or support service during the previous 12 months. Utilization of respite care was greater among those who employed professional home care (57% vs 24% of those without professional home care). Marked cantonal differences were also observed in utilization of respite care. The primary reason for not using respite services was “no demand” (80% of non-users of respite services), mainly among caregivers who were less emotionally affected by their caregiving tasks.

**Conclusions:**

Utilization of respite and support services depends more on place of residence and use of home care services than on functional status of the care recipient. Accordingly, programs should be tailored to the cultural context of their potential users. This is best achieved through coordination with local health care professionals who can identify needs, provide information, initiate referrals, and integrate the care into a larger support plan.

**Supplementary Information:**

The online version contains supplementary material available at 10.1186/s12913-021-06651-6.

## Background

Informal care can have positive effects on the well-being of care recipients, such as individuals with spinal cord injury (SCI). It is care provided outside the context of formal employment regulations [1], in which family caregivers are mostly involved. They assume substantial responsibilities, such as nursing, emotional, and practical support [2, 3], which can have negative impacts on their lives [4]. Respite care offers caregivers a temporary break from their daily routine and the stress from caregiving [5]. Utilization of respite care has been found to be generally low in different countries despite high levels of need [6]. To design appropriate respite care programs, it is necessary to identify beneficiary subgroups for different types of services [6, 7].

SCI represents a complex physical condition that requires long-term care responsibility, especially from family caregivers. Caregiving for persons with SCI can start at early age due to traumatic causes [8] and the family caregivers are younger compared to caregivers for other conditions [3, 8]. Given the long-term nature, family caregivers of persons with SCI bear high level of burden [4, 9], even when home nursing services are in place [10]. Temporary relief and support are crucial for the family caregivers to sustain their care. The use of respite and supporting programs is a topic yet to explore for caregivers for persons with SCI. As the care provision is widely different in amount and the tasks that they assisted [8], the family caregivers of persons with SCI can represent beneficiaries of services in different scenarios in caregiving at home.

Although emerging evidence was generated on respite care use, most studies focused on factors closely related to the caregiving situation. Caregivers’ cultural understanding of health care system, care tasks, and caregiving load were found associated with the use [5, 11, 12]. Other factors include caregivers’ income and education, as these competencies can facilitate the understanding of health care and the financing of care. However, as respite care is not confined to health care components, it is necessary to take account of broader contextual factors. A qualitative study in Switzerland [13] reported that the living situation and social participation can be attributable to maintaining the caregiver’s support network and further strengthen their access to relevant information and services. A systematic review [14] identified rurality as a barrier to respite service utilization, which, however, still needs a comprehensive view of socioeconomic geography of the locations. The same review also suggested the importance of differentiating types of service. Before the association between the influential factors and the utilization can be examined, a comprehensive exploration is needed to identify which predictors can be influential.

On that basis, the approach developed here addresses the identifying needs for a wider range of predictors and more service-specific reporting for different types of respite services. Regression modeling as commonly applied does not suffice to identify subgroups for the present purposes. In regression models, a linear relationship is assumed, only additive effects are detected for average members of a population, and there is a risk of overfitting when considering many predictors. The options for modeling non-linear relationships are limited, and the interactions between predictors are difficult to interpret [15]. To overcome these limitations, predictors of respite and support utilization were identified using classification and regression trees (CART) [15].

In this study, SCI in Switzerland was used as a case in point of respite use of family caregivers. In Switzerland, professional home care, the nursing service at care recipients’ homes, is covered by social health insurance. A bundle of health and social services currently fall into the umbrella category of relief and crisis support, which the present study included as respite care. This encompasses institutional and community-based services that take over caregiving responsibilities, provide timeout and information, and help develop social connections [16]. Following a decentralized organization, the services are provided by regions of residency (cantons), municipalities, professional care providers (home care and nursing home), and non-profit organizations [16]. Financing of respite care varies across providers.

This study aimed to quantify the uptake of different respite and support services for family caregivers, the reasons for non-use, and to explore the respective predictors. Specifically, the study aimed to 1) identify the utilization of various respite services and the characteristics of their main beneficiaries among family caregivers and 2) investigate the reasons for the non-use of respite services and the respective predictors.

## Methods

### Data collection

A cross-sectional survey of family caregivers for persons with SCI was conducted nationwide in Switzerland [17]. As contact information of family caregivers was not available, the study took a convenience sample of family caregiver nested in a national cohort study of persons with SCI. Between August 2016 and July 2017, the persons with SCI were contacted and asked to forward a questionnaire to their primary family caregiver. Their contact information was acquired from the Swiss Spinal Cord Injury Cohort Study (SwiSCI) database [18], which represents one of the largest community database in the context of SCI [19]. The inclusion criteria for persons with SCI for the present study followed those of SwiSCI, focusing on persons who had a chronic SCI for more than 2 years, were aged over 16 years, and resided in Switzerland. The exclusion criteria were persons with SCI resulting from congenital conditions, new injury in end-of-life care, or neurodegenerative disorders. Persons with SCI who reported not having a family caregiver were excluded. “Family caregiver” was defined as a partner, direct relative, or relative-in-law who cared for or assisted the person with SCI in daily living at home. The inclusion criteria for family caregivers were age over 18 years and ability to fill out the questionnaire in one of the survey languages (German, French, or Italian).

### Questionnaire and variables

The family caregivers were asked whether they used any of the listed 11 respite and support services over the previous 12 months. The use was defined and coded binary (1 = use and 0 = non-use). The services were retrieved using the term “relief service” from services provided by cantons, municipalities, and the Swiss Red Cross. These included caregiving services (home-based or institution-based caregiving for the care recipient), daily living support (driving services for the care recipient, household chores), support in crisis (emergency call system), information and knowledge transfer (advice and training), and social connections (companion and support groups). Although expanding beyond usual definitions of respite care, all of these services were included in the data analyses because they all provided the caregivers with relief during caregiving. These services are referred to as respite and support hereafter, or respite for short. For those who indicated that they had not used any respite care service, the reasons were classified among eight categories with multiple answers possible. The outcomes of reason mentioning were coded binary (1 = participant mentioned the reason and 0 = participant did not mention the reason).

To explore predictors in the multifold lives of the caregivers, the analyses included 128 potential predictors of different types related to socio-demographic characteristics, living situation, employment, finances, and caregiving situation of the caregiver and the care recipient. To facilitate comparison with the general population, the questionnaire largely utilized measurements used in population-based survey. Questions about satisfaction, interpersonal relationships, and leisure activities were measured using items with Likert-scales from the Swiss Household Panel [20]. The caregiver’s quality of life (QoL) was measured with a single item about overall quality of life (WHOQOL-BREF) [21]. The caregivers’ subjective social status was assessed by a single-item measure from the MacArthur Scale of Subjective Social Status [22]. Twelve items from the COPE-index [23] were used to capture perceived positive and negative aspects of caregiving. Psychometric properties of the instruments were evaluated in their respective sources.

### Data analysis

Odds ratios for respite use were calculated between participant groups that were hypothesized to have higher or lower needs for respite. Need for respite was expressed as caregiving burden, operationalized as caregiver’s QoL, care recipient’s wheelchair dependency, and caregiver’s time investment in care.

The method of classification tree was used to identify population subgroups with high or low percentages of respite outcomes. Each predictor included was tested to see if it can be divided into subgroups with significantly higher percentages of observations with respective outcome. A strength of this approach is that it allows for the identification of nested subgroups and nonlinear relationships between the outcomes and its predictors. The depth of the subgroups to be identified was determined by cross-validating the risk of misclassification.

Separate classification trees were built for each of the 11 types of respite and for the use of any respite or support as outcomes. Another set of classification trees was built for the eight reasons for non-use of respite as outcomes. In each classification tree, 128 potential predictors were included. The characteristics of the predictors were detailed in Supplementary Table 1, including the types and classes for binary and categorical predictors. The results are summarized as tables showing the most predictive subgroups for the respective outcomes. Data were prepared using Stata 16 for Windows (College Station, TX, USA). Classification trees were built with the R package “rpart” [24]. Missing records were omitted in the construction of classification trees. Given the exploratory nature of the study and the need to avoid over-fitting, missing values were not imputed. An evaluation of missing values was performed.

## Results

### Sample characteristics

The level of missing values was 5% for outcomes of respite care utilization, and 2% for the outcomes of reasons. The evaluation revealed a low level of missing values in most of the predictors (3–12%). Predictors of care tasks (12–22%) and receipt of financial compensation (20%) had more missing values, as did the variable “onetime expenses due to care”, which contained 42% of missing values.

Among the 4502 invitations, 1259 persons with SCI did not have a family caregiver. Additionally, excluded were 532 persons who were ineligible on other criteria, 397 untraceable contacts, 110 participation refusals, and 1487 non-responses. A total of 717 questionnaires were returned (a response rate 31%), and of those, 679 (95%) answered the question about respite care. The primary family caregivers were mostly female (72%), and the average age was 57 years (Table 1). A majority (76%) were spouses of the person with SCI, and 84% lived in the same household as their care recipient. Median care duration was 9 years, with a median time investment of 12 h per week. About a third of participants reported having used insurance-covered professional home care, with a median use of 8 h per week.

**Table 1:** Characteristics of the family caregivers and the persons with spinal cord injury

Characteristics	Statistics ***N*** = 679
***Characteristics of the family caregiver***	
*Sex–n (%)*	
Male	188 (27.7)
Female	488 (71.9)
*Age in years–mean (std)*	57.3 (13.9)
*Language region–n (%)*	
German	498 (73.3)
French	146 (21.5)
Italian	28 (4.1)
*Relationship to the SCI person–n (%)*	
Spouse/life partner	517 (76.1)
Mother/father	89 (13.1)
Child	35 (5.2)
Sibling	19 (2.8)
Other relative	7 (1.0)
*Living in the same household with the person with SCI–n (%)*	572 (84.2)
*Perceived quality of life–n (%)*	
Very good	150 (22.1)
Good	372 (54.8)
Neither good nor bad	131 (19.3)
Bad	10 (1.5)
Very bad	3 (0.4)
***Characteristics of caregiving situation***	
*Used at least one kind of respite or support during the last 12 months–n (%)*	
Yes	239 (35.2)
No	440 (64.8)
*Duration since taking care in years–median (Q*_*25*_*–Q*_*75*_*)*	9 (4–19)
*Time investment in caregiving in hours per week–median (Q*_*25*_*–Q*_*75*_*)*	12 (5–30)
*Other informal caregivers involved–n (%)*	279 (41.1)
*Hired professional home care–n (%)*	230 (33.9)
*Hired hours of professional home care–median (Q*_*25*_*–Q*_*75*_*)*	8 (4–14)
***Characteristics of the person with SCI***	
*Sex–n (%)*	
Male	499 (73.5)
Female	176 (25.9)
*Age in years–mean (std)*	56.4 (16.2)
*Time since injury in years–median (Q*_*25*_*–Q*_*75*_*)*	14 (5–26)
*Type of SCI–n (%)*	
Paraplegic	417 (61.4)
Tetraplegic	216 (31.8)
Missing	46 (6.8)
*Wheelchair dependency– n (%)*	
Completely dependent on wheelchair	464 (68.3)
Able to stand	22 (3.2)
Partially able to walk	166 (24.4)

The numbers of missing values are less than 5% if not specified otherwise

Quality of life was measured with a single item about overall quality of life (WHOQOL-BREF)

*Abbreviations*: *SCI* Spinal cord injury, *std*. Standard deviation, *Q*_*25*_ Lower quartile, *Q*_*75*_ Upper quartile

### Utilization of respite and predictors of utilization

Respite use was found to be higher among participants who reported a low QoL (OR 1.55, 95% CI 1.06 to 2.26), whose care recipient was wheelchair dependent (OR 1.72, 95% CI 1.18 to 2.49), and who reported a greater time investment in care provision (OR 2.34, 95% CI 1.69 to 3.25) (Table 2). About a third reported having used at least one type of respite care service during the previous 12 months (Table 3, graphic presentation in Additional File 2). The most commonly used service was a driving service for care recipients (16%), followed by household support (14%). Support groups and training courses were used by the fewest participants (1% in both cases).

**Table 2:** Correlation between need for respite and respite use

Need for respite indicated by burden	Odds ratio
Caregiver’s perceived quality of life	1.55 (1.06–2.26)*
Wheelchair dependency	1.72 (1.18–2.49)**
Time investment in care	2.34 (1.69–3.25)***

Odds ratios were calculated based on simple logistic regression. Respite care use: 0 = used none; 1 = used at least one kind of service.

Caregiver’s perceived quality of life (measured with overall quality of life in WHOQOL-BREF): 0 = high quality of life (very good, good); 1 = low quality of life (very bad, bad, neither good nor bad).

Wheelchair dependency: 0 = not fully wheelchair dependent (able to stand, able to walk); 1 = fully wheelchair dependent.

Time investment in care: 0 = low investment (< sample median 12 h/week); 1 = high investment (≥ sample median 12 h/week).

* = *p* < 0.05, ** = *p* < 0.01, *** = *p* < 0.001

**Table 3:** Predictors of utilization of respite services during the last 12 months

Respite Service ***N*** = 679 n (%)	Typical users			Typical non-users		
	Predictors	N^b^	n (%)^c^	Predictors	N^b^	n (%)^c^
**Has used at least one kind of respite care** **239 (35.2%)**	→Received professional home care	230	132 (57%)^a^	→Did not receive professional home care	449	107 (24%)^a^
	→Had onetime expense due to care over 22,000 CHF	16	16 (100%)	→Care service was not considered an important information topic	428	92 (21%)
***Types of services***						
**Driving service** **108 (15.9%)**	→Received over 1.5 h/week of professional home care	220	64 (29%)	→Received less than 1.5 h/week of professional home care	459	44 (1%)^a^
	→Family caregiver lived in canton of ZH, ZG, BS, BL, SG, TI, GE	76	33 (43%)			
	→Person with SCI injured less than 7 years ago	25	17 (68%)^a^			
**Household support** **96 (14.1%)**	→Received over 0.75 h/week of professional home care	214	59 (28%)	→Received less 0.75 h/week of professional home care	465	37 (8%)^a^
	→Lived in canton of UR, ZG, TI	16	12 (75%)^a^	→Family caregiver lived in other cantons than SO, BL and VD	397	23 (6%)
**Relief offer for holidays/short term home care** **49 (7.2%)**	→Received professional home care	230	36 (16%)	→Did not receive professional home care	449	13 (3%)^a^
	→Lived in canton of ZH, BE, SH, SG, GR, TG	96	27 (28%)			
	→Family caregiver assisted in washing face and hands	32	14 (44%)			
**Emergency call** **38 (5.6%)**	→Family caregivers aged 72 years old or older	107	16 (15%)	→Family caregiver younger than 72 years	572	22 (4%)^a^
	→Lived in canton of SZ, TI, VD, GE	22	10 (45%)	→Family caregiver did not assist in mobility in the house	515	15 (3%)
	→Family caregiver assisted in mobility in moderate distance	10	8 (80%)^a^	→Family caregiver lived in other cantons than ZG, FR, SO, TG, TI, VD and VS	351	3 (1%)
**Advice** **30 (4.4%)**	No predictor identified	–	–	No predictor identified	–	–
**Respite assistance at home during the day** **29 (4.3%)**	→Care service was considered an important information topic	88	15 (17%)	→Care service was not considered an important information topic	591	14 (2%)^a^
	→Lived in canton of ZH, SG, GR, AG, GE	26	9 (35%)			
	→Received financial compensation for caregiving	17	9 (53%)^a^			
**Day care in nursing home** **27 (4.0%)**	→Family caregiver aged 67 years or older	197	19 (10%)	→Family caregiver younger than 67 years old	482	8 (2%)^a^
	→Family caregiver lived in canton of SG, GR, NE	11	6 (55%)^a^			
**Night care** **16 (2.4%)**	No predictor identified	–	–	No predictor identified	–	–
**Social companionship/visit** **16 (2.4%)**	→Family caregiver missed someone to talk to	129	10 (8%)	→Family caregiver did not miss someone to talk to	550	6 (1%)^a^
	→Family caregiver lived in canton of SO, TI, VD and JU	23	6 (26%)			
	→Family caregiver did not assist in foot washing	11	6 (55%)^a^			
**Support groups for family members** **7 (1.0%)**	No predictor identified	–	–	No predictor identified	–	–
**Training courses** **6 (0.9%)**	No predictor identified	–	–	No predictor identified	–	–

*Abbreviations*: *SCI* Spinal cord injury, *CHF* Swiss Francs, Cantons were presented in abbreviations

^a^ The predictor significantly predicted the outcome

^b^ N = the total number of participants in the respective nodes

^c^ n = the number of participants who utilized a particular service among the participants in the respective nodes; % = the percentage of participants utilizing a particular service in the respective nodes

Reported overall utilization of respite was higher among participants with professional home care (57% vs 24% among those without professional home care). Professional home care was also found associated with specific types of respite or support services, including driving services (29% vs 1% among those with less than 1.5 weekly hours of professional home care), household support (28% vs 8% among those with less than 0.75 weekly hours of professional home care), and relief offers for holidays (16% vs 3% among those without professional home care). The strongest determinants of respite utilization were utilization of professional home care, either as binary predictor or as continuous predictor of the hired hours, and canton of residence (Supplementary Table 2). They were the primary predictors of the utilization of five types of respite care services and of the overall utilization of respite care. Receiving financial compensation was the most decisive factor for using daytime respite services. Use of daytime respite was the only outcome related to financing. Whether the family caregiver assumed the care tasks of foot washing was the most decisive for social companionship. Several predictors related to caregiving tasks appeared to have predictive power for using particular types of services. In general, family caregivers’ time investment in care was a weak predictor of respite care utilization, as were the care recipient’s functional status, caregiver’s satisfaction with their financial situation, and income.

### Reasons for not utilizing respite and predictors

The most common reason for non-utilization of respite care services was “no demand”, specified by 80% of the 432 non-users (Table 4, graphic presentation in Additional File 2). Other reasons were “sufficient support by family or friends” (22%) and “uncomfortableness with strangers/family preference” (12%). Less common reasons included costs (9%), availability (3%), scheduling (2%), bad experiences (1%), and mistrust (1%).

**Table 4:** Predictors of reasons for non-utilization of respite services during the previous 12 months

Situation for non-use of respite services ***N*** = 432 n (%)	Reason endorsed			Reason not endorsed		
	Predictors	N^b^	n (%)^c^	Predictors	N^b^	n (%)^c^
**No demand 344 (79.6%)**	→Sometimes/never perceived caregiving as negative to their emotional well-being	402	336 (84%)^a^	→Mostly/always perceived caregiving as negative to their emotional wellbeing	30	8 (27%)^a^
	→Highly satisfied with their financial situation	261	238 (91%)	→Lived in canton of FR, SG, AG, TI, VD, VS, NE, GE	15	0 (0%)
	→Family caregiver spent less than 52 h/week in caregiving	249	231 (93%)			
**Sufficient support by family or friends 94 (21.8%)**	→Other informal caregiver involved	180	62 (34%)	→No other informal caregiver involved	252	32 (13%)^a^
	→Family caregiver lived in canton of ZH, BE, LU, SZ, NW, GL, ZG, FR, BL, SG, GR, AG, TI, VD, VS, NE	160	62 (39%)	→Family caregiver lived in other cantons than SH, GR, TI, VS, NE	216	20 (9%)
	→Lower personal income (less than 6000 CHF per month)	64	35 (55%)^a^	→Family caregiver with low satisfaction of interpersonal relationship	156	9 (6%)
**Care recipient uncomfortable with strangers/preference of family 53 (12.3%)**	→Family caregiver spent 30 h/week or more in caregiving	91	28 (31%)	→Family caregiver spent less than 30 h/week in caregiving	341	25 (7%)^a^
	→Lived in canton of LU, SZ, OW, GL, FR, GR, AG, TI, VS, GE	36	20 (56%)^a^	→Sometimes/never perceived caregiving as negative to their emotional well-being	322	18 (6%)
	→Family caregiver aged 54 years or older	29	20 (69%)	→Family caregiver did not assist in dressing lower body	231	6 (3%)
**Too expensive/not covered by insurance** **38 (8.8%)**	→Low quality of life (“very bad” to “neither good nor bad”)	79	20 (25%)	→High quality of life (“good” to “very good”)	353	18 (5%)^a^
	→Live in canton of LU, SH, SG, AG, GE	18	11 (61%)^a^			
**Not available** **13 (3.0%)**	No predictor identified	–	–	No predictor identified	–	–
**Inconvenient schedule** **9 (2.1%)**	→Family caregiver spent 65 h/week or more in caregiving	21	3 (14%)	→Family caregiver spent less than 65 h/week in caregiving	411	6 (1%)^a^
	→Lived in canton of GL, FR, BL	5	3 (60%)^a^	→Family caregiver did not live alone	383	3 (1%)
**Bad experience with service provider** **6 (1.4%)**	No predictor identified	–	–	No predictor identified	–	–
**No trust in service providers** **3 (0.7%)**	No predictor identified	–	–	No predictor identified	–	–

*Abbreviations*: *SCI* Spinal cord injury; Cantons were presented in abbreviations

^a^ The predictor significantly predicted the outcome

^b^ *N* = the total number of participants in the respective nodes

^c^ *n* = the number of participants who utilized a particular service among the participants in the respective nodes; % = the percentage of utilizing a particular service in the respective nodes

The reason of “no demand” for respite services primarily related to less negative impact of caregiving on the caregiver’s emotional well-being and to higher satisfaction with their financial situation. The vast majority (91%) of the participants with these characteristics reported not having a need for respite care, and this group constitutes 55% of the total non-user group. Participants who were negatively affected by caregiving and did not mention “no demand” for respite care were geographically clustered. Half of them lived in eight—mainly French-speaking—cantons (out of 26) and referred to barriers other than “no demand”. Family caregivers who invested more time in care more often cited the care recipient’s preference for family caregiver as a reason for not using respite services. The canton of residence was the most important factor in delineating the various reasons for non-utilization of respite care services (Supplementary Table 3).

Using cross-validation, only two outcomes–overall utilization of respite and reason of no demand–could be predicted with significantly lower risk of misclassification. If a participant used professional home care, utilization of respite care could be predicted. If a participant reported low negative emotional impact of caregiving, this participant was likely to indicate no demand for respite.

## Discussion

The present study highlights the influence of contextual factors in utilization of respite care and variation across different types of services. Adding to the current evidence, which mainly focused on caregivers of frail older adults and persons with dementia [5], these results illustrated respite care use among caregivers for persons with long-term physical limitations. The current caregiver sample assumed more responsibilities in physical and medical care, compared to the general caregiver population in Switzerland [17]. Yet training was not among the most commonly used support, which is thought to facilitate coping with complex caregiving situation. Instead, more users were found in services that support daily living and household. Participants in the current study had much longer care duration, compared to the median duration of 5 years in the general Swiss caregiver population [25]. It is possible that they have passed the phase of coping and established their contact with professional support, which the current cross-sectional study could not capture. Although mostly non-significant, the predictors of utilization of different services were quite diverse, indicating the importance of service-specific attention to different groups of caregivers and care recipients [14]. For instance, older family caregivers could benefit more from support of emergency call and day care for care recipients. Potential users of different services should be proactively identified and provided with assistance to access relevant services.

Regional variability was the most decisive determinant of respite use and for the reasons for non-utilization. Similarly, health care use in Switzerland is linked to the cantonal supply of services and financing [26]. Households with persons with SCI tend to be clustered in cantons where the SCI-specific services are well established [27]. However, participants in the present study rarely mentioned unavailability as a reason for non-use. The use did not seem to distinguish between urban and rural areas, contrasting a previous study in US [28]. It is possible that the service structure is not substantially different between urban and rural areas in Switzerland. Although financial support varies across cantons [16], this did not seem to be a key factor, as only a few participants mentioned cost as a barrier to access respite care services, contrary to a previous study conducted in Australia [11]. Cultural characteristics, as described by the language region, did not fully explain the unequal local utilization of respite care services, although health care use varies across the different language regions in Switzerland [29]. As there are no national regulations for respite care, other local characteristics may be more influential. As found in studies about caregivers of persons with dementia, the caregivers and their immediate environment might have passive views and cultural understanding towards their use of respite, assuming the needs of the care recipients have priority [6, 30]. In Switzerland, higher population density was positively linked to use of general health care, creating fewer social barriers to seeking professional support [26]. One study in the Netherlands suggested that close contacts and shared responsibility in the neighborhood could be valuable assets for service provision for informal caregivers. In-depth studies are needed to clarify which local characteristics can enhance the access to respite and support for family caregivers.

Family caregivers who employed professional home care were more likely to use respite care services. It is difficult to link these two through the families’ financial capacity, as predictors related to financing only appeared influential for one type of services. The findings seemed contradictory across services, which indicates type-specific attention for financing. The predictive power of caregiver burden on respite care utilization was small, though other studies have identified this as a strong determinant [31]. The use of home nursing has been associated with a higher physical dependency [26]. The results showed that utilization of service clustered among caregivers with high service demand, indicating that these family caregivers seem to need both sources of support. The present findings also confirm earlier evidence that professional home care is the main source of temporary breaks and condition-specific consultations for family caregivers [16].

It was not surprising that the most common reason for non-utilization was “no demand”. The statement of “no demand” was linked to the subjective perception of burden. Similar to the findings regarding the care of persons with dementia, care for the affected person was the main consideration when deciding about utilization, and family caregivers rarely declared their need for a break [6]. Family caregivers may place more emphasis on the needs of the care recipient, feel guilty about being away, consider the caregiving an expectation of being a family member [32, 33]; they may wish to maintain a positive aspect, such as their close relationship with the care recipient [34]. Even when most family caregivers wish to continue to provide care themselves, they trust health professionals as a reliable source of information and acknowledge their central role in encouraging caregivers to use external support [34]. Family caregivers could benefit from encouragement and active assistance to find respite care, especially from local health professionals [35]. Because they are in close contact with the families, health professionals can identify needs, if not explicitly expressed by the family caregivers, and provide information or initiate referrals. The study results also indicate a need to look beyond the objective burden, given that the care load was not linked to the topic of demand. The perceived subjective burden, however, is less visible. The results indicated a greater need for support among those with low QoL. It is therefore recommended that this indicator should be included in clinical or routine assessments so that services can be initiated for those who are in need. The assessment can be conducted by home care nursing professionals.

### Limitations

The study had two limitations. First, the low response rate of the survey calls representativeness of the target population into question. In comparison to other studies of care provision for persons with SCI, the current study represented family caregivers with rather low time investment in care [17]. The questionnaire contained items addressing all aspects of the caregiver’s life, which may be unduly time consuming for family caregivers to answer. It might be a hurdle for family caregivers with heavy care load to participate [17], so that they might be underrepresented in the sample, leading to underrepresented prediction power of care load. As lack of time was a barrier to access services [31], this group of caregivers still need more proactive support. Additionally, the questionnaire was forwarded by persons with SCI, whose self-definition of a caregiver may exclude family members who were seen as “helping out”–for example, by performing mainly household–rather than a caregiver.

A second limitation is that missing values of predictors could not be accounted for. This may lead to weakened predictive power of certain factors, as the algorithm will search for the next best surrogate predictor. However, as the proportion of missing values was acceptable, this is likely to have had only a minor impact on the results. The overall predictive power of a predictor still reflected the variable importance. The current study serves as an exploration of potential predictors. Follow-up research based on more rigorous modeling is needed.

## Conclusion

Applying classification trees, the current study identified that use of respite and support was primarily determined by contextual factors. Utilization of respite depends more on place of residence and use of home care services than on the functional status of the care recipient. The findings invite further investigation of characteristics of home care professionals and local context that can facilitate the use of respite and support. More attention should also be devoted to family caregivers’ subjective burden, which is less visible than their objective burden, in order to provide more proactive assessment or support. The respite programs should be adapted to the cultural, regional, and personal contexts of their intended users. This is best achieved in coordination with local health care professionals who can identify needs, provide individual information, initiate referrals, and integrate care activities into a larger support plan.

## Supplementary Information

**Additional file 1.** The file contains three supplementary tables. The first one lists all the predictors included in the data analyses. The second and the third list the most important predictors for each outcome.

**Additional file 2.** Results of classification trees presented in tree graphs. The file contains the graphs of each classification tree, for which at least one predictor could be identified.

**Additional file 3.** Questionnaire (English translation). The file contains the questionnaire, from which data of the present study were retrieved. It refers to an English translation of the original questionnaire.

## Data Availability

The datasets used and/or analyzed during the current study are available from the corresponding author on reasonable request.

## Abbreviations

CART: Classification and Regression Tree
COPE-index: Carers of Older People in Europe Index
CHF: Swiss Francs
QoL: Quality of Life
SCI: Spinal Cord Injury
Std: Standard Deviation
SwiSCI: Swiss Spinal Cord Injury Cohort Study
Q_25_: Lower quartile
Q_75_: Upper quartile
WHOQOL-BREF: World Health Organization Quality of Life Brief Version

## Swiss cantons

AG: Aargau (Argovia)
AI: Appenzell Innerrhoden (Inner Rhodes)
AR: Appenzell Ausserrhoden (Outer Rhodes)
BE: Berne (Bern)
BL: Basel-Landschaft (Basle-Country)
BS: Basel-Stadt (Basle-City)
FR: Fribourg
GE: Geneva
GL: Glarus
GR: Graubünden (Grisons)
JU: Jura
LU: Lucerne
NE: Neuchâtel
NW: Nidwalden (Nidwald)
OW: Obwalden (Obwald)
SG: St. Gallen (St. Gall)
SH: Schaffhausen
SO: Solothurn
SZ: Schwyz
TG: Thurgau (Thurgovia)
TI: Ticino
UR: Uri
VD: Vaud
VS: Valais
ZG: Zug
ZH: Zürich (Zurich)
