# Supplementary material for: Who are the beneficiaries and what are the reasons for non-utilization of care respite and support services? A cross-sectional study on family caregivers
Source: BMC Health Serv Res. 2021 Jul 2;21:637. doi: 10.1186/s12913-021-06651-6 (PMC8254343; doi:10.1186/s12913-021-06651-6)
Supplement: Supplementary file 1 — Additional file 1. The file contains three supplementary tables. The first one lists all the predictors included in the data analyses. The second and the third list the most important predictors for each outcome. [file 12913_2021_6651_MOESM1_ESM.docx]

Supplementary Table 1 List of predictors used in the analysis on utilization of respite services

| **Predictors** | **Predictor Type** | **Classes** | **Variable names** |
| --- | --- | --- | --- |
| Sex of the caregiver | binary | Male/female | ic_pc_sex |
| Age of the caregiver | continuous |  | ic_pc_age |
| Caregiver’s migration background | binary | Swiss born/immigrant | ic_pc_migr |
| Canton of living | nominal |  | ic_pc_canton_liv |
| Language region | nominal | German/French/Italian | language_region |
| Marital status | binary | Married or registered partnership/others | ic_pc_marital_status |
| Education | ordinal | Did not finish mandatory education/mandatory education/secondary II/tertiary | ic_pc_education |
| Personal monthly income | ordinal |  | ic_pc_income_personal |
| Household monthly income | ordinal |  | ic_pc_income_household |
| Health insurance type of the caregiver | nominal | General/half private/private/don’t know | ic_pc_health_insurance |
| Caregiver lives alone | binary | Yes/no | ic_ls_household_member_1 |
| Child(ren) under 14 years of age in the household | binary | Yes/no | ic_ls_household_member_2 |
| Co-habitant(s) aged 15–64 years in the household | binary | Yes/no | ic_ls_household_member_3 |
| Co-habitant(s) above 65 years old in the household | binary | Yes/no | ic_ls_household_member_4 |
| Family caregivers cohabits with the person with SCI | binary | Yes/no | ic_ls_same_household |
| Travel time to care recipient | ordinal |  | ic_ls_distance |
| Relationship to the person with SCI | nominal | Spouse or partner/child/sibling/parent/other | ic_ls_care_relation |
| Satisfaction with own health (caregiver) | ordinal | 0–10 | ic_ls_health_sat |
| Quality of life | ordinal |  | ic_ls_qual_life |
| Employment | binary | Employed/not employed | employed |
| Full-time employment | binary | Full-time employed/part-time employed | fulltime |
| Satisfaction with financial situation | ordinal | 0–10 | ic_vc_finances_sat |
| Out-of-pocket expenditure | binary | Yes/no | ic_fi_care_expenses |
| Out-of-pocket expenditure monthly in CHF | continuous |  | ic_fi_care_expenses_month |
| Out-of-pocket expenditure once in CHF | continuous |  | ic_fi_care_expenses_once |
| Duration of caregiving in years | continuous |  | ic_sn_care_year |
| Caregiving tasks^1^ | binary | Yes/no | care_fam_1–care_fam_21 |
| Family caregiver’s total time investment in care per week | continuous | hrs/week | ic_sn_effort_care_num |
| Other family members involved | binary | Yes/no | involved |
| Professional home care hired | binary | Yes/no | ic_sn_daily_support_6 |
| Hired hours of professional home care | continuous |  | ic_sn_daily_support_hours |
| Subjective perception of impact of the caregiving on one’s life^2^ | ordinal | Never/sometime/mostly/always | ic_sn_affect_soul–ic_sn_affect_support |
| Primary contact for health problem^3^ | binary | Yes/no | pc_1–pc_6 |
| Caregiver ever sought information | binary | Yes/no | ic_info_seek |
| Topic of information sought^4^ | binary | Yes/no | ic_info_source_1–ic_info_source_15 |
| Source of information sought^5^ | binary | Yes/no | ic_info_used_1–ic_info_used_14 |
| Close relationship for personal contact | ordinal | Several persons/one person/no one/don’t know | ic_ir_relation_personal |
| Family caregivers missed someone to talk to | ordinal | Yes/no/don’t know | ic_ir_relation_missing |
| Satisfaction with interpersonal relationship | ordinal | 0–10 | ic_ir_relation_sat |
| Caregiver received financial compensation | binary | Yes/no | ic_su_compensation |
| Leisure activities and social life^6^ | ordinal | Daily/once a week/once a month/once a year/never | ic_sl_activity_friends–ic_sl_activity_religion |
| Numbers of vacations without the person with SCI | continuous |  | ic_sl_holidays_num |
| Subjective social status | ordinal | 0–10 | ic_soc_subjective_pos |
| Rurality of residence region | ordinal | Urban/suburb/rural | Urban |
| Sex of the person with SCI | binary | Male/female | ic_ps_sex |
| Age of the person with SCI | continuous |  | ic_ps_age |
| Type of SCI | binary | Paraplegic/tetraplegic | ic_sci_type |
| Cause of SCI | nominal | Accident/disease/other | ic_sci_cause |
| Wheelchair dependency | ordinal | Dependent on wheelchair/able to stand/partly able to walk | ic_sci_ability |
| Duration in years since injury | continuous |  | ic_sci_length |
| Person with SCI being employed | binary | Yes/no | ic_ps_workstatus_1 |
| Person with SCI received financial support^7^ | binary | Yes/no | ic_ps_financial_support_1–ic_ps_financial_support_5 |

^1^ In total there are 21 different tasks, asking whether the caregiver assist in a certain activity of daily living, such as eating and drinking, washing face and hand, washing upper body etc.

^2^ The subjective perception of impact of caregiving was elaborated with 12 items, including perceived negative to one’s well-being, too demanding, negative to one’s physical health, negative to family, trapped in the role of caregivers, negative to one’s relationship with friends, a worthwhile task, good relationship with care recipient, being valued as a caregiver, managed the well as caregivers, perceived sufficient support from health services, and perceived as supported in general.

^3^ Primary contacts for health problem included 6 items, including general practitioner, SCI specialist, professional home care, SCI nursing consultants, general hospital, SCI specialist clinic

^4^ In total there were 15 topic areas concerning life with SCI, five out of which the participants mentioned as the most needed information topics, including health-related, fitness, technology, finances, caregiving, house modification, accommodation for persons with SCI, legal issues, sexuality, employment, psychological problems, and social relationship.

^5^ In total there were 14 information sources, three out of which the participants mentioned as the most used information sources, including SCI specialist, general practitioner, other health professionals, SCI nursing consultants, social counselling, research institute, SCI patient association, support group, peer with SCI, family/friend/colleague, online media, TV/radio, print media, and literature on the topic.

^6^ Leisure and social life contains 10 items on leisure activities and social participation, such as meeting friends, sport events, participation in association/club etc.

^7^ The financial support consists of 5 items on compensation for the person with SCI, such as helplessness allowance, compensation for caregiving etc.

Supplementary Table 2 Most important predictors for utilization of respite services

| Respite care service | Most important predictor | Reduced impurity in % ^*^ |
| --- | --- | --- |
| Has used at least one kind of respite service | Professional home care hired | 19% |
| Driving service | Hired hours of professional home care | 14% |
| Household support | Canton of living (family caregivers) | 19% |
| Relief offer for holidays/short term home care | Hired hours of professional home care | 13% |
| Emergency call | Canton of living (family caregivers) | 22% |
| Advice | No predictor identified | - |
| Respite assistance at home during the day | Caregiver received financial compensation for the caregiving | 26% |
| Day care in nursing home | Canton of living (family caregivers) | 25% |
| Night care | No predictor identified | - |
| Social companionship/visit | Family caregiver assisted in foot washing | 22% |
| Support groups for family members | No predictor identified | - |
| Training | No predictor identified | - |

* Reduced impurity was calculated based on how much the split improves the prediction of outcome, by reducing impurity. The percentages represent a proportion of impurity reduced by the respective variable, out of the total combined reduced impurity of all variables in the tree.

Supplementary Table 3 Most important predictors for the reasons of non-utilization of respite services

| **Reasons for non-utilization of respite services** | **Most important predictor** | **Reduced impurity in % ^*^** |
| --- | --- | --- |
| No demand | Family caregiver perceived negative impact of caregiving on emotional well-being | 16% |
| Sufficient support by family or friends | Canton of living (family caregiver) | 21% |
| Care recipient uncomfortable with strangers/preference of family | Canton of living (family caregiver) | 15% |
| Too expensive/not covered by insurance | Canton of living (family caregiver) | 45% |
| Service not available | No predictor identified | - |
| Inconvenient schedule | Canton of living (family caregiver) | 56% |
| Bad experience with service provider | No predictor identified | - |
| No trust in service providers | No predictor identified | - |

* Reduced impurity was calculated based on how much the split improves the prediction of outcome, by reducing impurity. The percentages represent a proportion of impurity reduced by the respective variable, out of the total combined reduced impurity of all variables in the tree.
