# Supplementary material for: Who are the beneficiaries and what are the reasons for non-utilization of care respite and support services? A cross-sectional study on family caregivers
Source: BMC Health Serv Res. 2021 Jul 2;21:637. doi: 10.1186/s12913-021-06651-6 (PMC8254343; doi:10.1186/s12913-021-06651-6)

UTILIZATION OF RESPITE CARE AND PREDICTORS

Use of at least one type of respite care

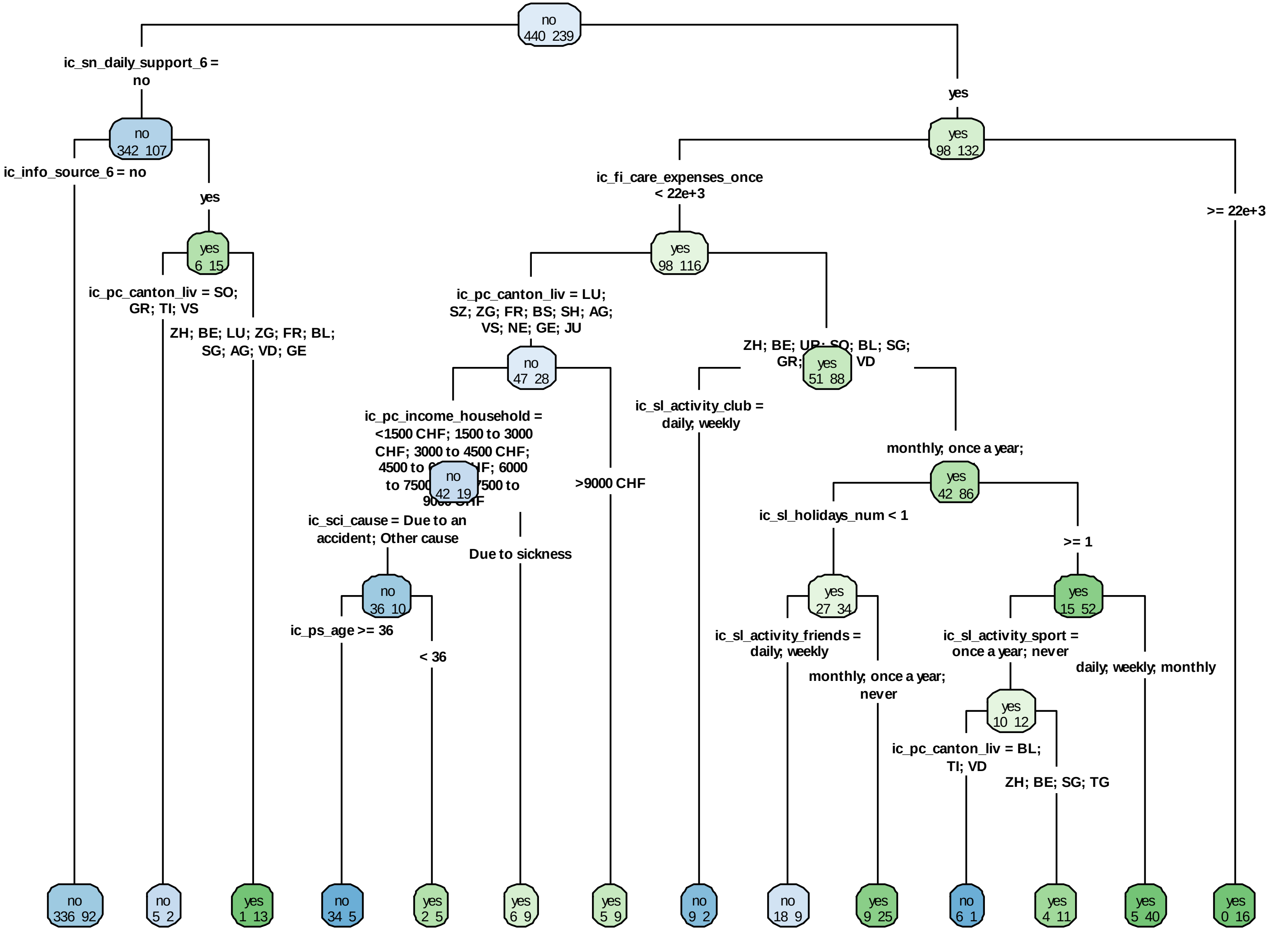

Use of at least one type of respite (pruned)

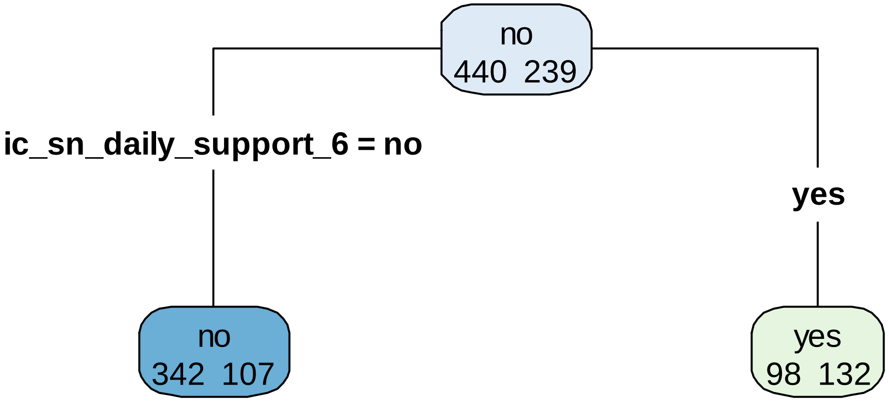

Use of driving service

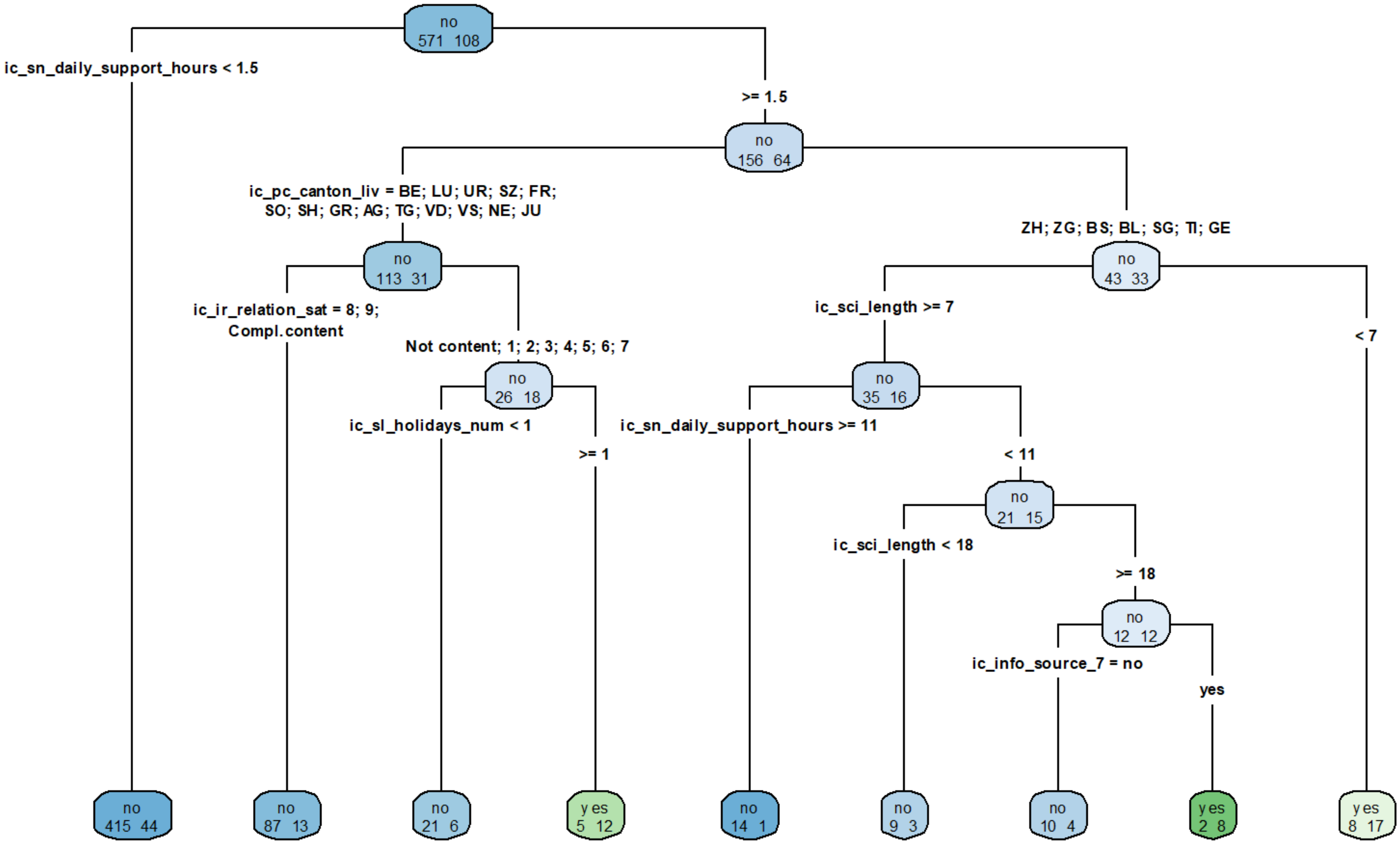

Use of household support

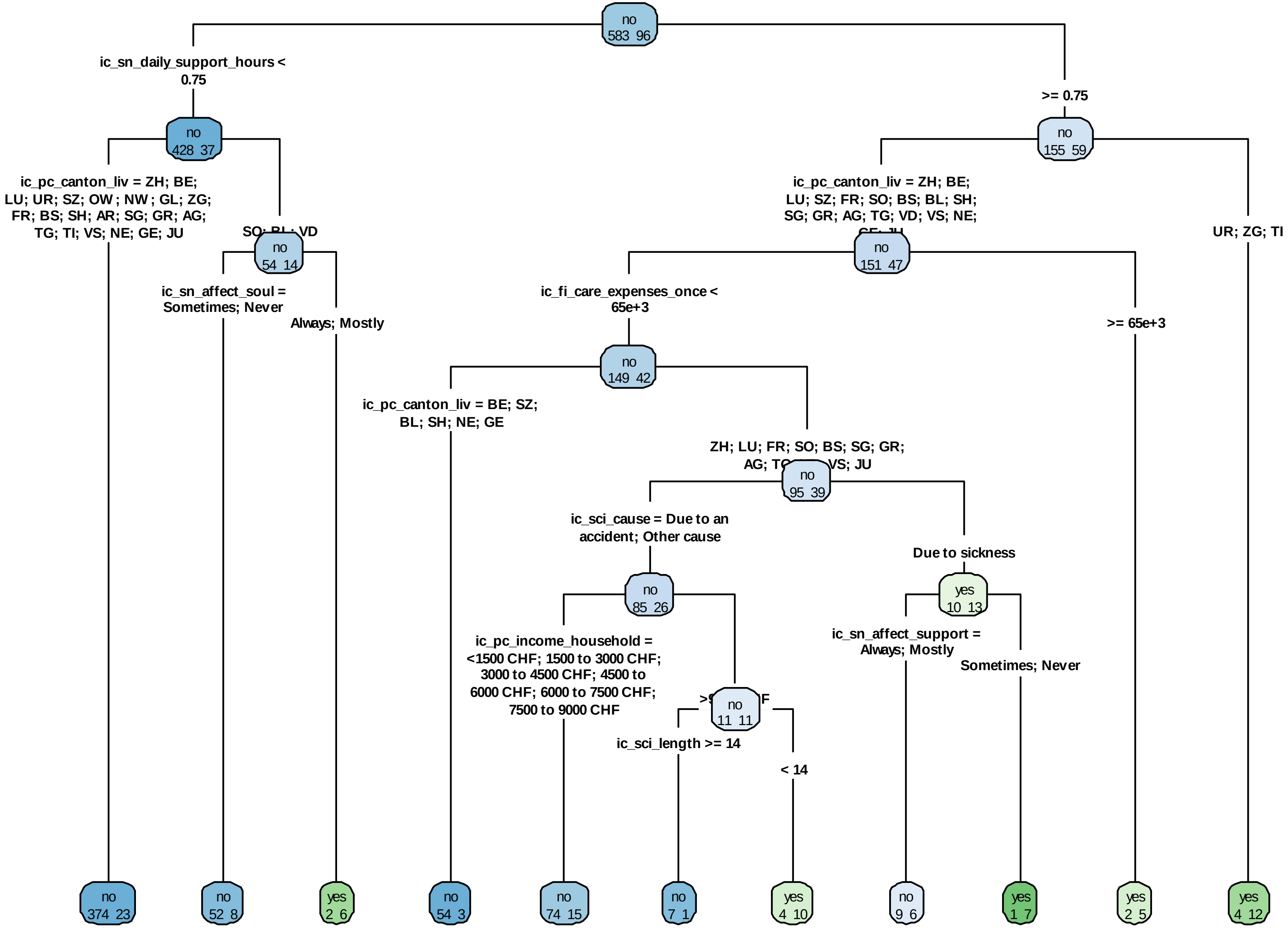

Use of relief for holidays or short-term home care

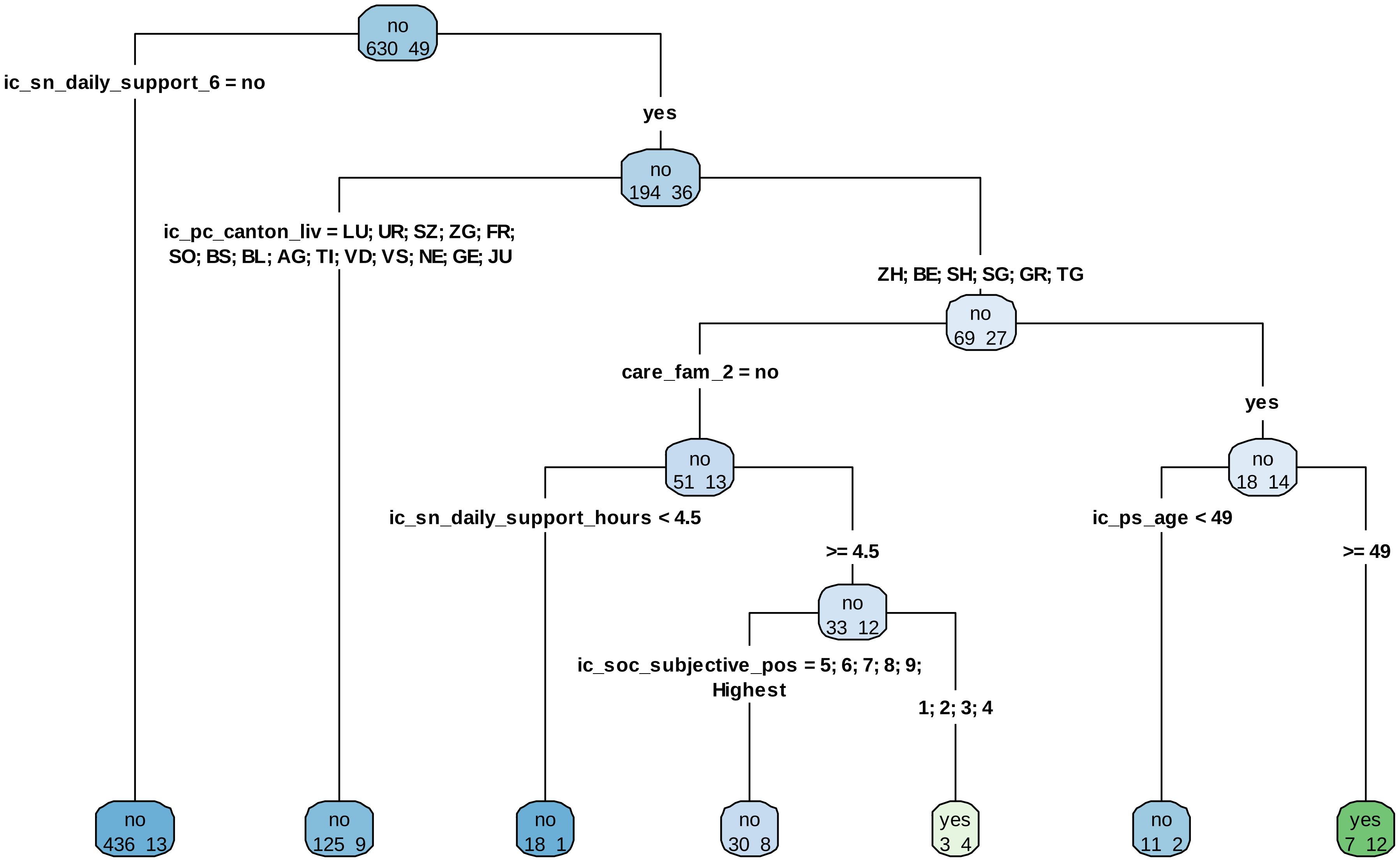

Use of emergency call

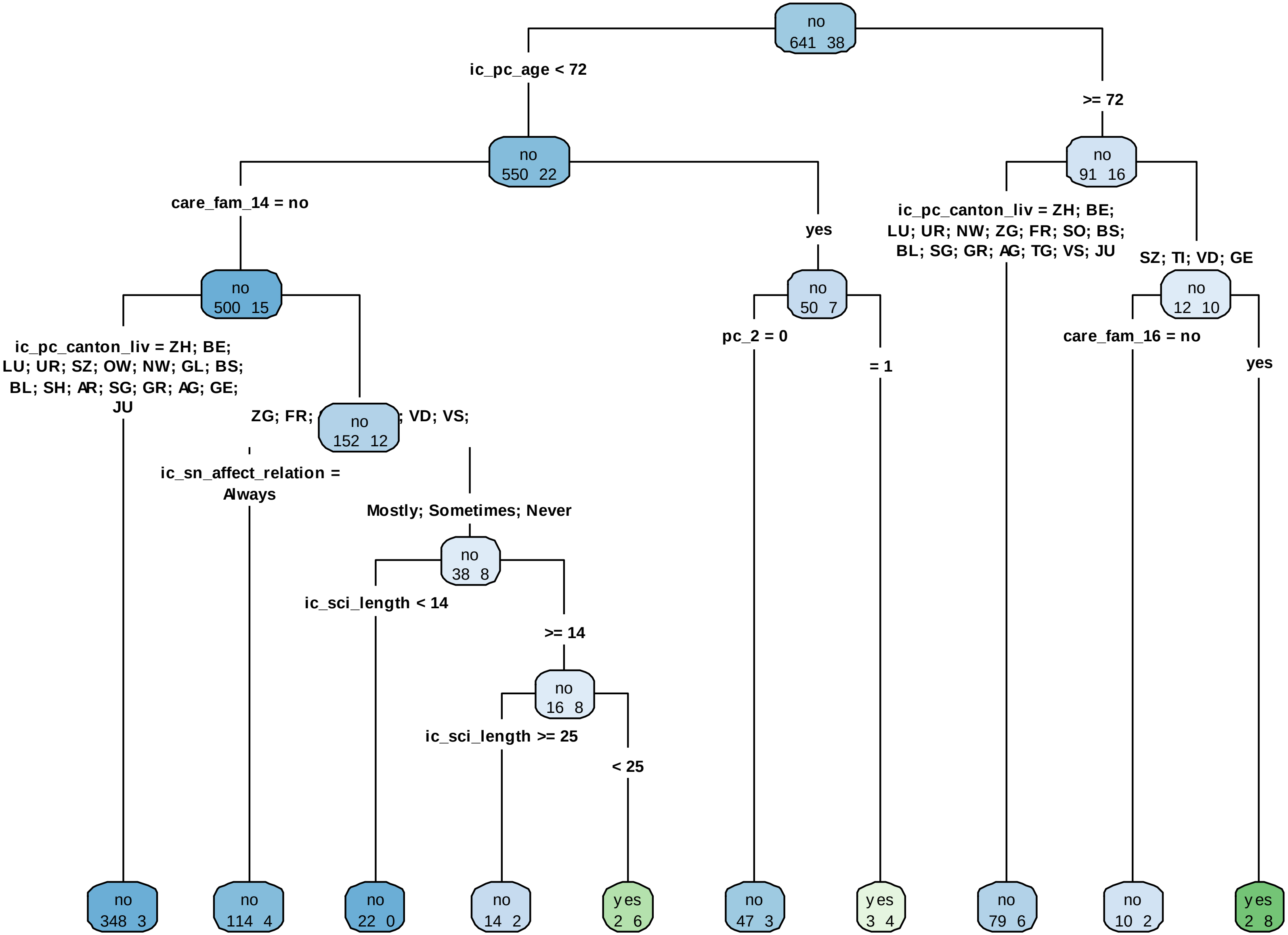

Use of respite assistance at home during the day

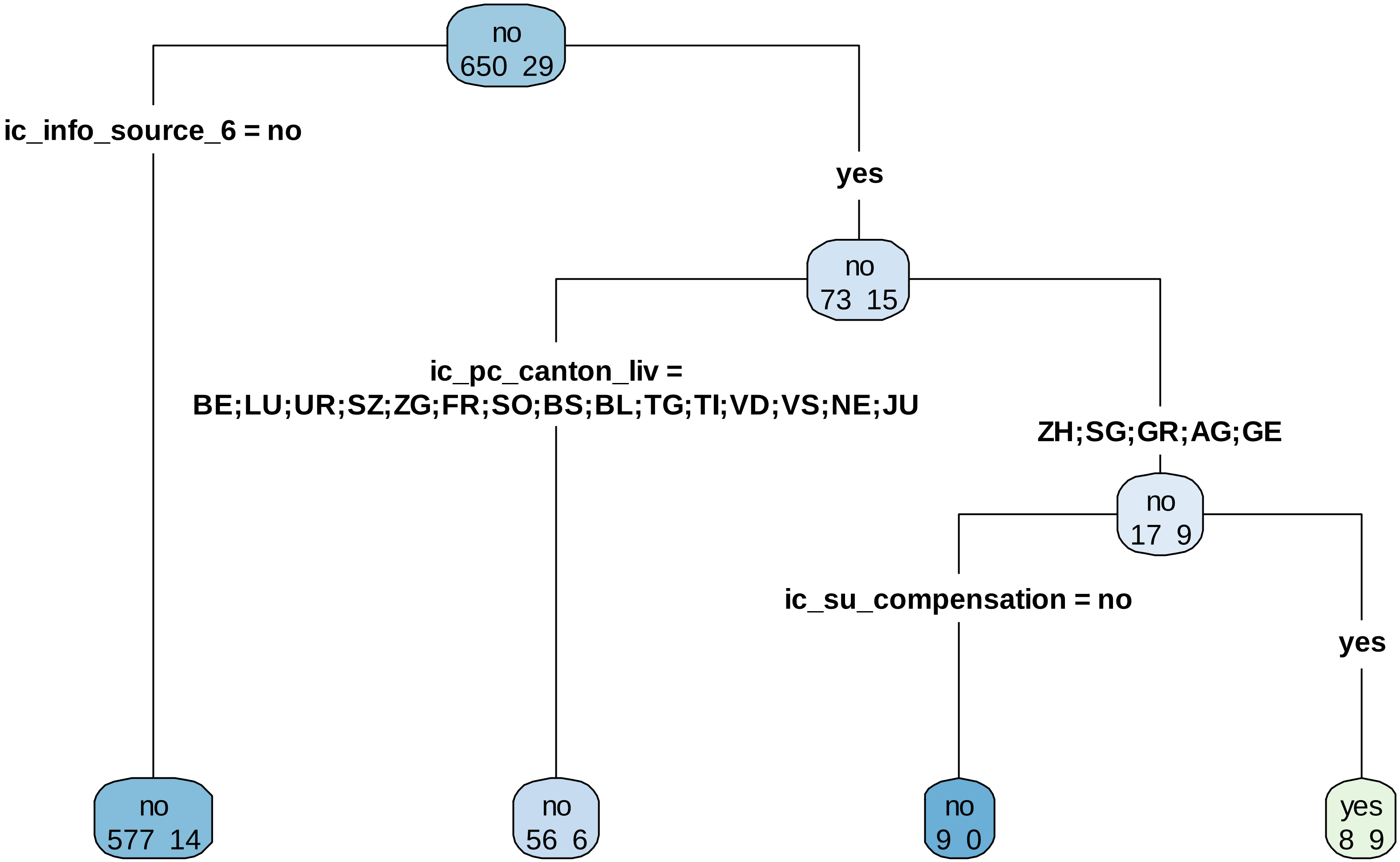

Use of day care in nursing homes

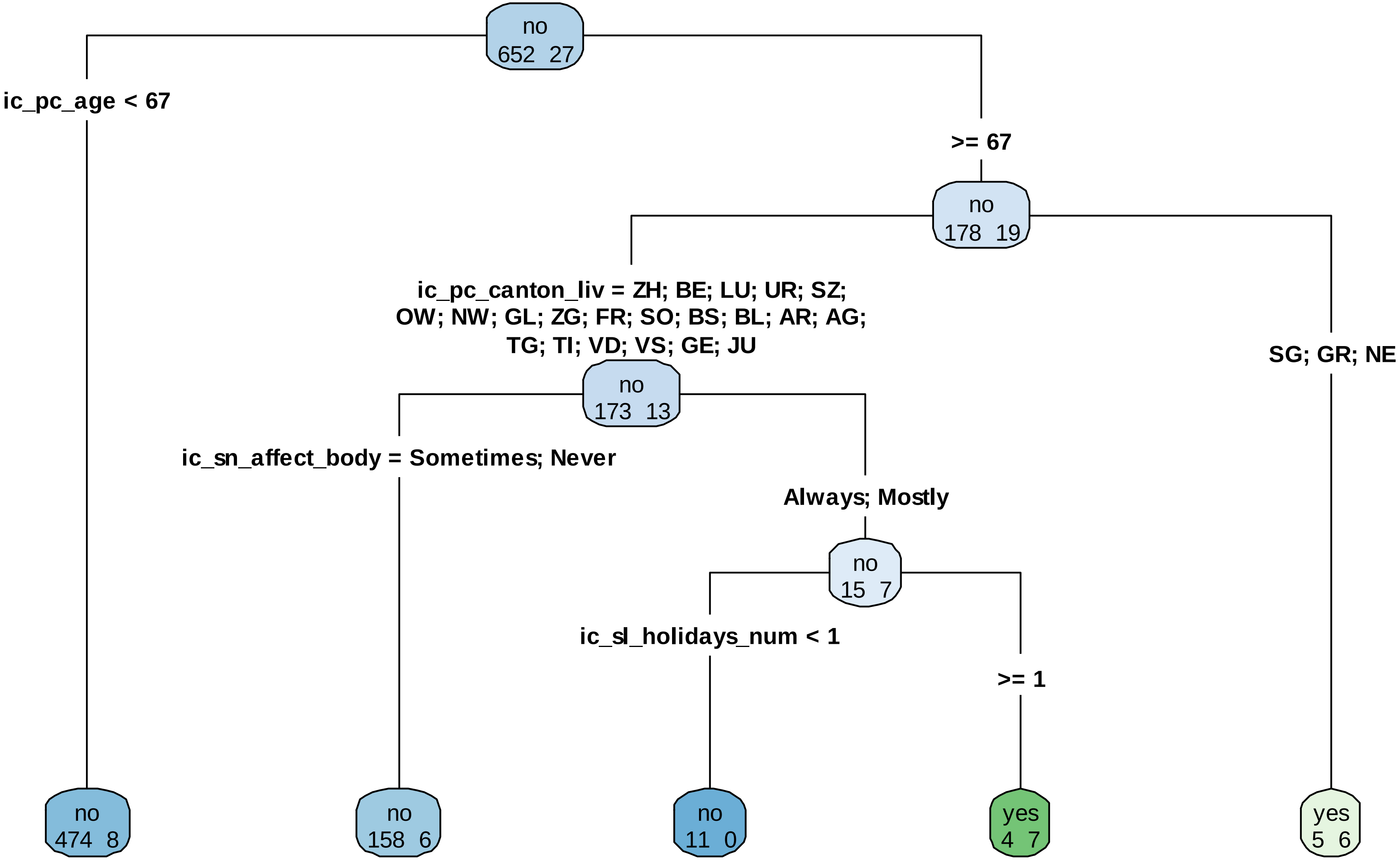

Use of social companion or visits

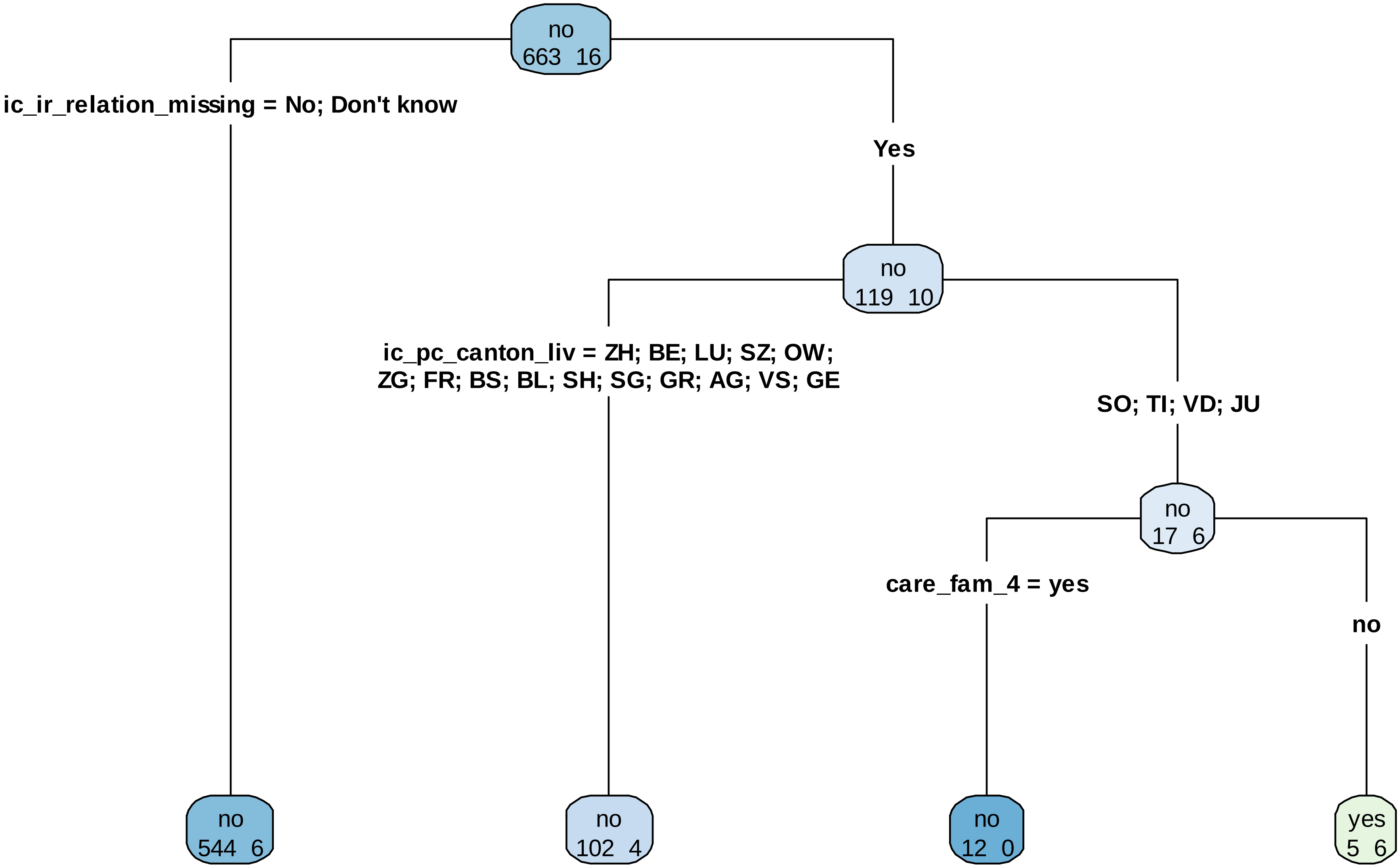

REASONS FOR NON-USE AND PREDICTORS

Non-use because of no demand

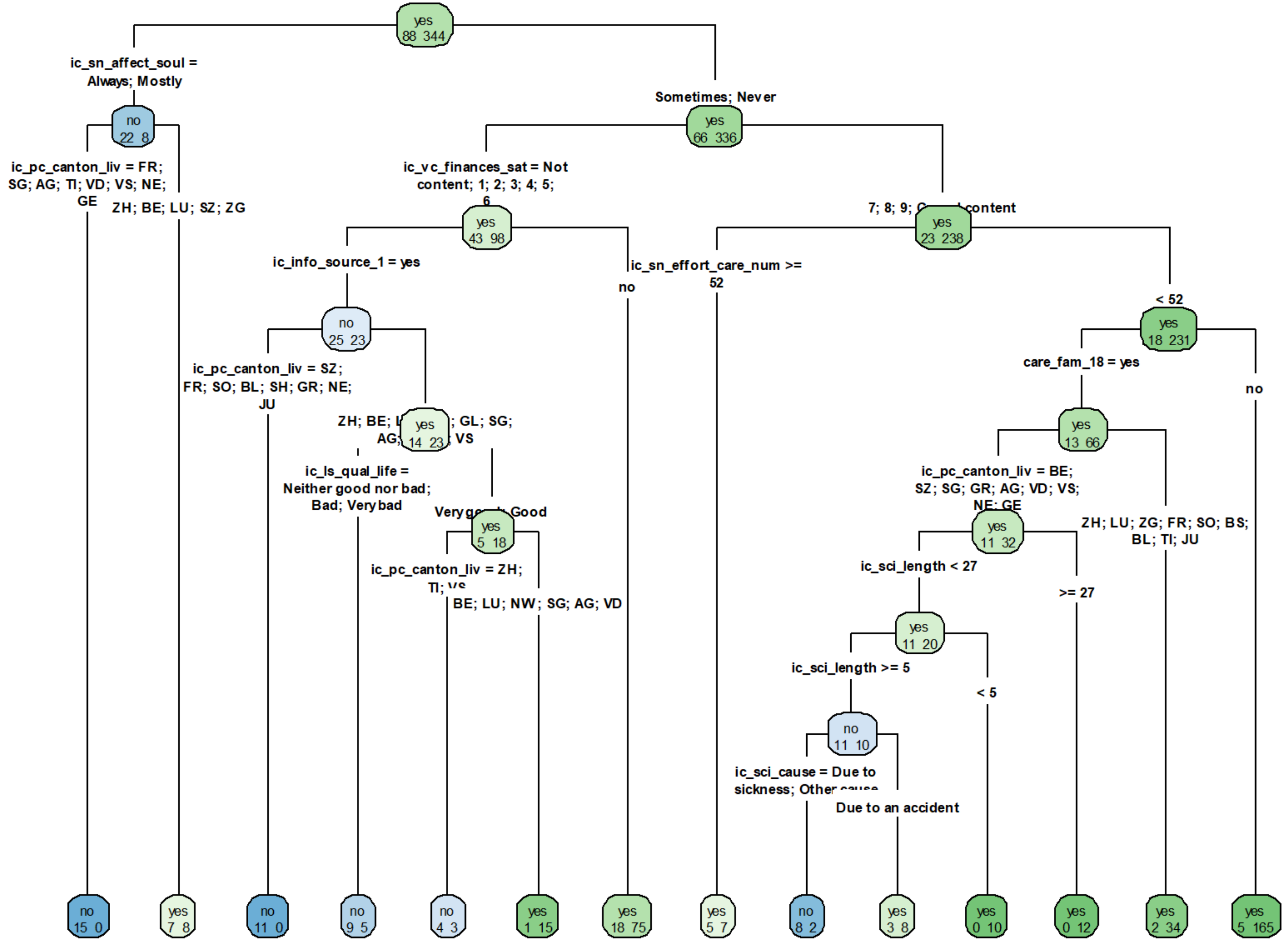

Non-use because of no demand (pruned)

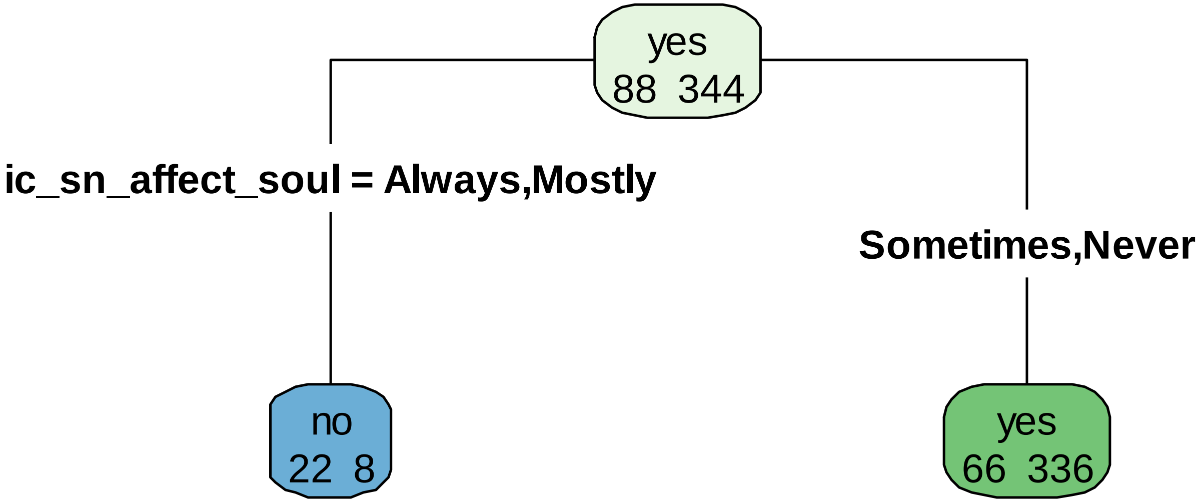

Non-use because of sufficient support by friends and family

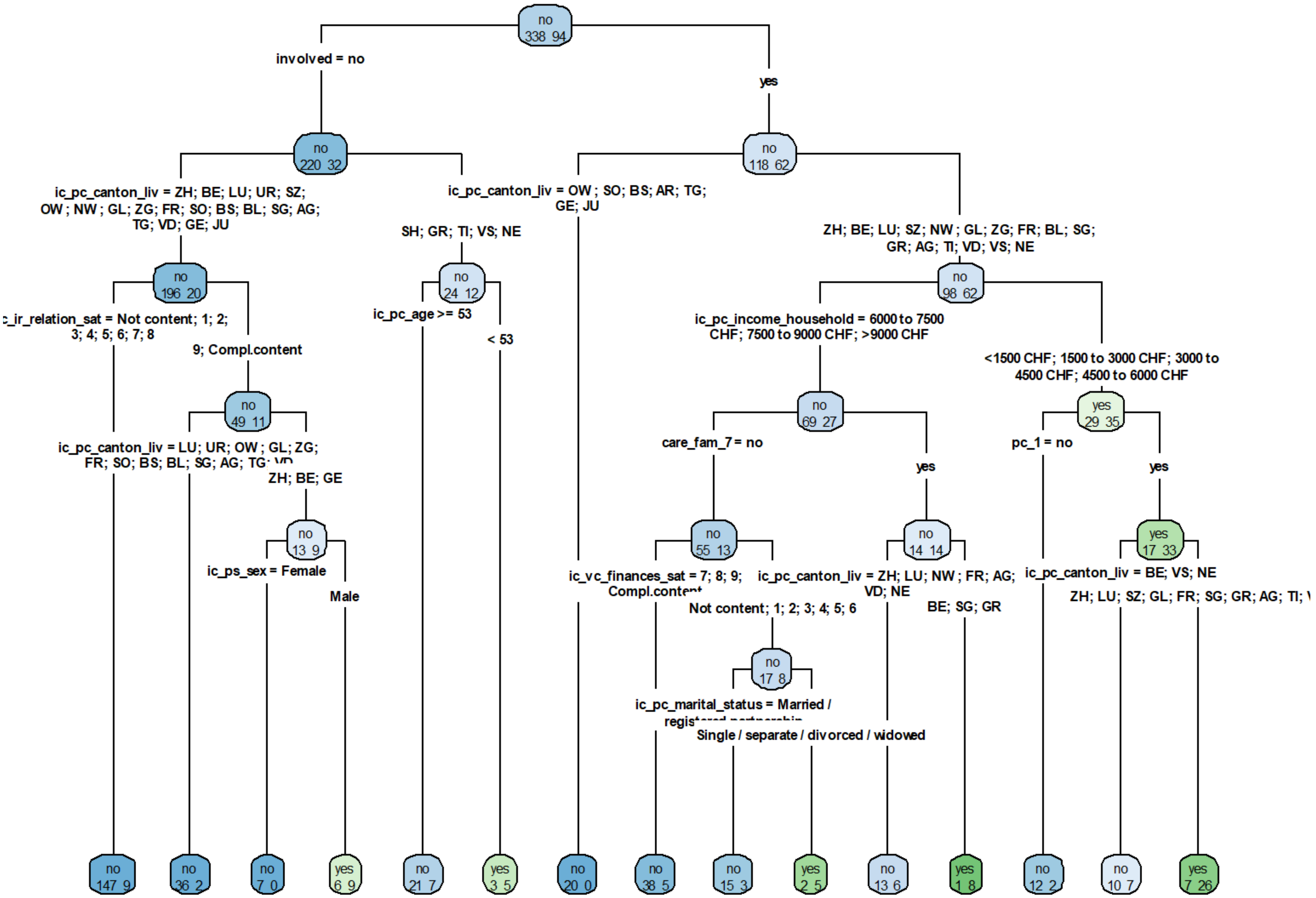

Non-use because of care recipient’s uncomfortableness or family value

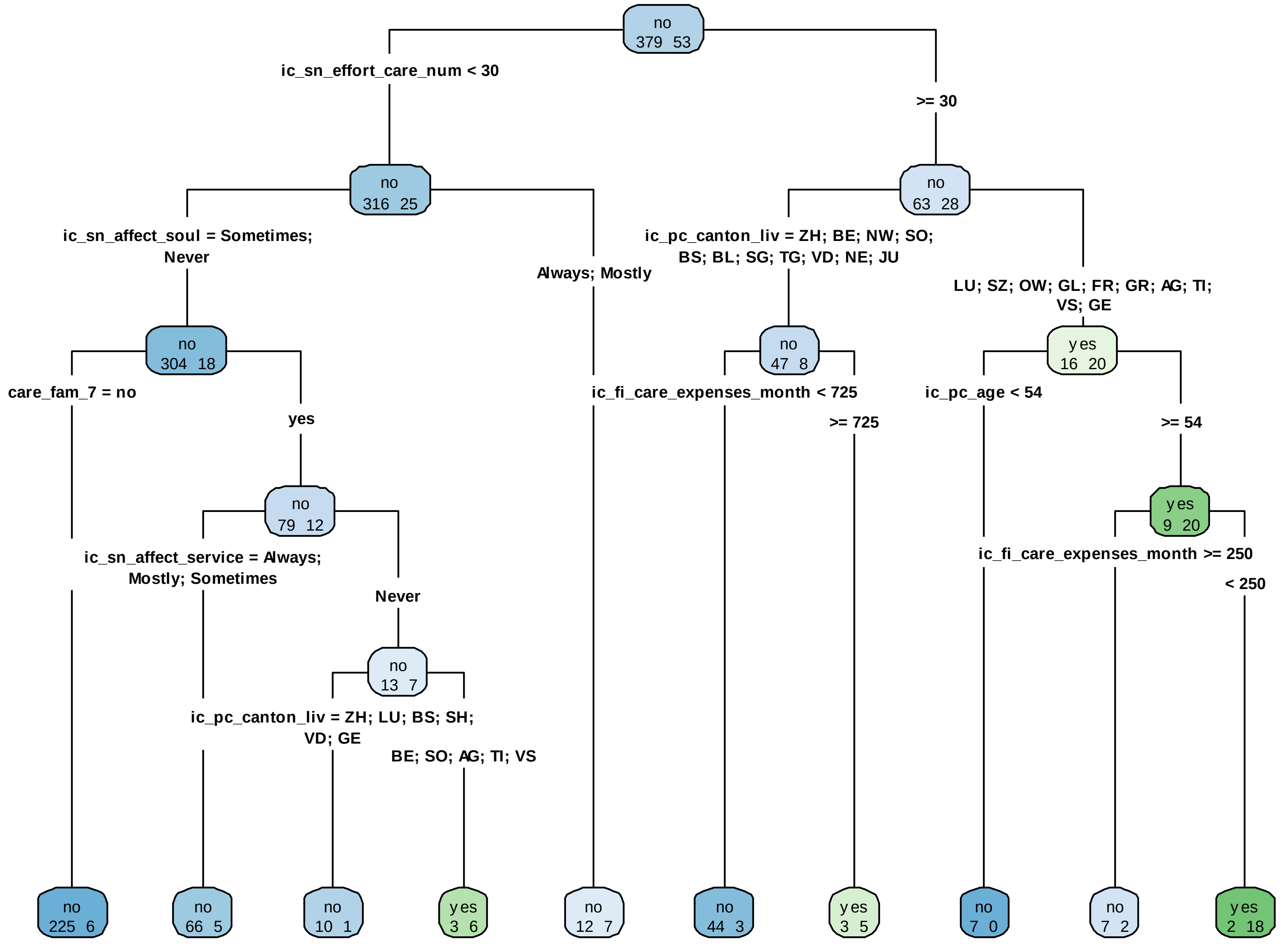

Non-use because of cost

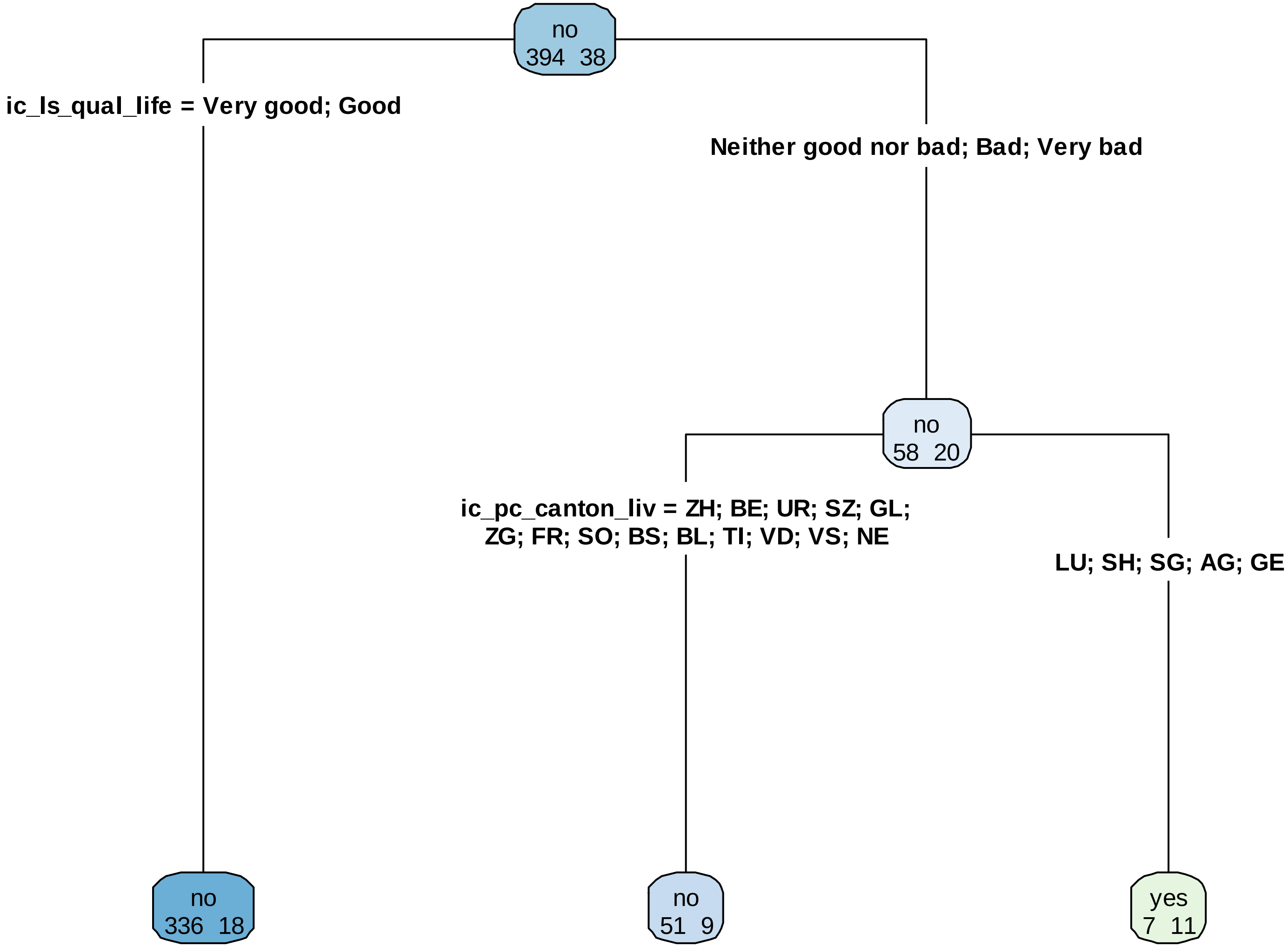

Non-use because of inconvenient Schedule

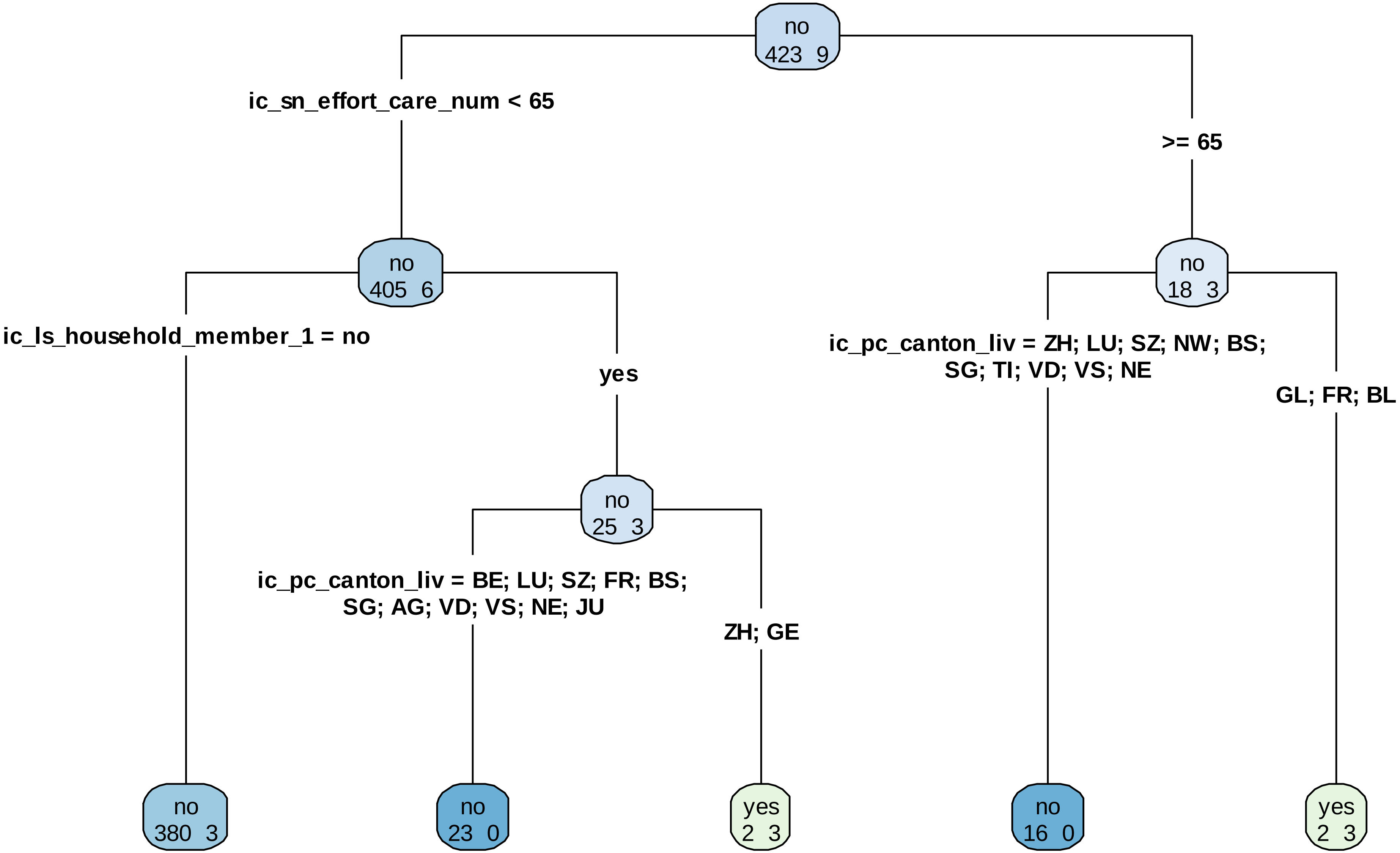

Supplement: Supplementary file 2 — Additional file 2. Results of classification trees presented in tree graphs. The file contains the graphs of each classification tree, for which at least one predictor could be identified. [file 12913_2021_6651_MOESM2_ESM.pdf]
