# Supplementary material for: Who are the beneficiaries and what are the reasons for non-utilization of care respite and support services? A cross-sectional study on family caregivers
Source: BMC Health Serv Res. 2021 Jul 2;21:637. doi: 10.1186/s12913-021-06651-6 (PMC8254343; doi:10.1186/s12913-021-06651-6)
Supplement: Supplementary file 3 — Additional file 3. Questionnaire (English translation). The file contains the questionnaire, from which data of the present study were retrieved. It refers to an English translation of the original questionnaire. [file 12913_2021_6651_MOESM3_ESM.pdf]

Informal Care by Family Members for Persons with Spinal Cord Injury Questionnaire

Personal information

In the following questions, we would like to ask you about personal situation

1. **Please indicate your sex**

   - ○ Male
   - ○ Female

2. **Please indicate the year in which you were born**

   ✐............... please indicate the year (e.g.1976)

3. **What is your nationality?**

   Check all that apply

   - ☐ Swiss
   - ☐ Non-Swiss

4. **How many years have you lived in Switzerland?**

   - ○ Have lived in Switzerland from birth onwards
   - ○ Have lived in Switzerland since ✐............... (please indicate the year, e.g. 2001)
   - ○ Not applicable, I live in another country:

     ✐............... (please indicate the country, and to Q6)

5. **Please indicate the canton in which you are currently living**

   - ○ AG Argau
   - ○ AI Appenzell I. Rh.
   - ○ AR Appenzell A. Rh.
   - ○ BE Bern
   - ○ BS Basel Stadt
   - ○ BL Basel Land
   - ○ FR Freibourg
   - ○ GE Genf
   - ○ GL Glarus
   - ○ GR Graubuenden
   - ○ JU Jura
   - ○ LU Luzern
   - ○ NE Neuenburg
   - ○ NW Nidwalden
   - ○ OW Obwalden
   - ○ SG St. Gallen
   - ○ SH Schaffhausen
   - ○ SO Solothurn

- ○ SZ Schwyz
- ○ TG Thurgau
- ○ TI Tessin
- ○ UR Uri
- ○ VD Waadt
- ○ VS Wallis
- ○ ZG Zug
- ○ ZH Zürich

6. What is your current marital status?

- ○ Married/cohabiting
- ○ Single/divorced/widowed

7. Which is the highest level of education achieved

- ○ No schooling or incomplete compulsory school
- ○ Completed compulsory school (lower secondary school or secondary level I)
- ○ Vocational training
- ○ University or higher

8. What is your monthly personal gross income?

- ○ Under CHF 1,500
- ○ CHF 1,500 to CHF 3,000
- ○ CHF 3,001 to CHF 4,500
- ○ CHF 4,501 to CHF 6,000
- ○ CHF 6,001 to CHF 7,500
- ○ CHF 7,501 to CHF 9,000
- ○ More than CHF 9,000

9. What is the monthly gross income of your household?

- ○ Under CHF 1,500
- ○ CHF 1,500 to CHF 3,000
- ○ CHF 3,001 to CHF 4,500
- ○ CHF 4,501 to CHF 6,000
- ○ CHF 6,001 to CHF 7,500
- ○ CHF 7,501 to CHF 9,000
- ○ More than CHF 9,000

10. Which type of health insurance do you have?

- ○ Basic health insurance
- ○ Semi-private health insurance
- ○ Private health insurance
- ○ Don’t know

Living situation, Quality of life

In the following questions, we would like to ask you about your living arrangements, home situation, and quality of life

11. **Who lives in your household besides you?**

Check all that apply

- ☐ Nobody, I live alone
- ☐ Children under 14 years of age, number: ✎...............
- ☐ Persons between 14 and 64 years of age, number: ✎...............
- ☐ Persons over 64 years of age, number: ✎...............

12. **Are you living in the same household with the person you take care of?**

- ○ Yes
- ○ No

13. **How much travel time do you need every time for one way when you travel to the person whom you take care of?**

- ○ None. I live in the same household
- ○ Less than 30 minutes
- ○ Between 30 minutes to 60 minutes
- ○ One hour
- ○ Two hours
- ○ More than two hours

14. **Relation with the person that you take care of?**

I am

- ○ Spouse of the person
- ○ Cohabiter/life partner of the person
- ○ Son/daughter of the person
- ○ Foster child of the person
- ○ Grandson/daughter of the person
- ○ Brother/sister of the person
- ○ Father/mother of the person
- ○ Grandfather/mother of the person
- ○ Son/daughter in law of the person
- ○ Father/mother in law of the person
- ○ Brother/sister in law of the person
- ○ Other relative

15. **How satisfied are you with your state of health, if 0 means "not at all satisfied" and 10 "completely satisfied"?**

	Not satisfied at all										Completely satisfied
	0	1	2	3	4	5	6	7	8	9	10
Satisfaction with my health	○	○	○	○	○	○	○	○	○	○	○

16. **How would you evaluate your general life quality?**

- ○ Very good
- ○ Good

- ○ Neither good nor bad
- ○ Bad
- ○ Very bad

Employment

In the following questions, we would like to ask you about your working situation.

17. What is your current employment status?

Check all that apply

- ☐ Full-time paid work
- ☐ Part-time paid work
- ☐ In school, training (apprentice, pupil, student)
- ☐ Work in protected atelier (for handicapped persons)
- ☐ Retired person (old-age) to Q26
- ☐ Other retired persons (invalidity, etc.) to Q26
- ☐ Unemployed to Q26
- ☐ Other situation, further education, non-paid leave

18. If working part-time what is your working quota?

✎.............. % working quota

19. What is your current occupation?

✎................ (name of your job)

20. Have you needed to be absent in your regular working hours because of caregiving?

- ○ Yes
- ○ No

If yes, which possibility of leaving do you have?

Check all that apply

- ☐ Sick leave
- ☐ Unpaid leave
- ☐ Flexible working time
- ☐ Compensated with overtime
- ☐ Holidays
- ☐ Others: ________________________

21. On a scale from 0 “not at all satisfied” to 10 “completely satisfied”, can you indicate your degree of satisfaction for your job in general?

	Not satisfied at all									Completely satisfied	N/A
	0	1	2	3	4	5	6	7	8	9	10	
Satisfaction with my job	○	○	○	○	○	○	○	○	○	○	○	○

22. Do you want to work more, less or the same amount of hours as you do today?

- ○ work more hours
- ○ work less hours
- ○ work the same amount of hours

23. Is there any change in your occupation since you started to take care of the person with SCI?

- ○ No change at all to Q26
- ○ Yes to Q24
- ○ Quitted my job to Q24

24. What was your working quota before?

✐.............. % working quota

25. What was your occupation before?

✐................ (name of your job)

26. Overall how satisfied are you with your financial situation, if 0 means "not at all satisfied" and 10 "completely satisfied"?

	Not satisfied at all									Completely satisfied
	0	1	2	3	4	5	6	7	8	9	10
Satisfaction with my financial situation	○	○	○	○	○	○	○	○	○	○	○

Financial needs

In the following questions, we would like to ask you about additional expenditures

27. Has caring resulted in any additional financial costs (out-of-pocket expenditures)?

- ○ No to Q29
- ○ Yes. I do have some extra expenses.

What were the expenses for?

Check all that apply

- ☐ Adaptation of the Home environment and or furniture
- ☐ Travel costs
- ☐ Special food
- ☐ Medicines
- ☐ Other, please specify ✐..............

28. What is your estimated amount of additional financial costs?

✐.............. Swiss Francs per month

And/or

✐.............. Swiss Francs once in total

Investment in Caregiving

This section is about your situation as a caregiver. We would like to learn from you how much you invest, what tasks you fulfil and what barriers you perceive.

29. Please indicate the year in which you first took over the caregiving

✐…………… (such as: 2011)

30. Were there any noteworthy interruptions since then?

○ No to Q32

○ Yes.

How many month in total.

✐…………… (number of months)

31. Please indicate the reasons for the interruptions

✐……………

32ff In what activities do you help the person with SCI and how many hours do you spend every week on them?

Please indicate how many hours you spent per week.

Not applicable means you do not assist in the activities

Activities	Numbers of hours per week	Not applicable
**32. Eating and drinking**	✐……………	○
**33. Washing face and hands**<br>Please think about your assistance in hand washing, face washing, tooth brushing, combing, shaving or make-up	✐……………	○
**34. Washing upper body and head**	✐……………	○
**35. Washing feet**	✐……………	○
**36. Washing lower body**	✐……………	○
**37. Dressing upper body**<br>Dressing the upper body includes putting on and taking off clothes like t-shirts, blouses, shirts, bras, shawls, or orthoses (e.g., arm splint, neck brace, and corset).	✐……………	○
**38. Dressing lower body**<br>Dressing the lower body includes putting on and taking off clothes like shorts, trousers, shoes, socks, belts, or orthoses (e.g., leg splint).	✐……………	○
***39.* Caregiving in relation to Respiration**	✐……………	○
**40. Bladder management and use of toilet**	✐……………	○
**41. Bowel management and use of toilet**	✐……………	○
**42. Transfer between bed and wheelchair**	✐……………	○
**43. Transfers from the wheelchair to the toilet/tub**	✐……………	○
**44. Climbing stairs**	✐……………	○

**45. Moving indoors**	✐……………	○
**46. Transfer between wheelchair and car**	✐……………	○
**47. Moving outdoors moderate distance (10 to 100 meter)**	✐……………	○
**48. Moving outdoors over 100 meter**	✐……………	○
**49. Company to therapy, doctor visits, and to other offices**	✐……………	○
**50. Housekeeping (cooking, tidying, laundry etc)**	✐……………	○
**51. (Grocery) shopping**	✐……………	○
**52. Paperwork (fill out forms, handling bills, bank or financial affairs)**	✐……………	○
**53. Others, please specify** ✐……………	✐……………	

54. How many hours in total do you spend on the caregiving?

✐…………… (number of hours per week)

55. Who else takes care of the person with spinal cord injury in the daily living?

Check all that apply

- ☐ Spouse/partner of the person
- ☐ Child(ren) of the person
- ☐ Parent(s) of the person
- ☐ Sibling(s) of the person
- ☐ Friend(s)/acquaintance(s)/neighbor(s) of the person
- ☐ Professional home care, hours per week ✐……………
- ☐ Other persons, namely ✐……………
- ☐ Other institutes ✐……………

56. To what extent can you be available for the person in case of need and showing understanding, by talking with the person for example, if 0 means "not at all" and 10 "a great deal"?

	Not at all									A great deal
	0	1	2	3	4	5	6	7	8	9	10
Available in case of need and understanding	○	○	○	○	○	○	○	○	○	○	○

57. If necessary, in your opinion, to what extent can you provide the person with useful advice, if 0 means "not at all" and 10 "a great deal"?

Please provide judgement of possible support, even when no support of such kind is needed now.

	Not at all										A great deal
	0	1	2	3	4	5	6	7	8	9	10
Useful advice	○	○	○	○	○	○	○	○	○	○	○

58ff Please indicate how providing care has affected your life.

	Always	Often	Some-times	Never
**58. Does caregiving have a negative effect on your emotional well-being?**	○	○	○	○
**59. Do you find caregiving too demanding?**	○	○	○	○
**60. Does caregiving have a negative effect on your physical health?**	○	○	○	○
**61. Does caregiving cause difficulties in your relationship with your family?**	○	○	○	○
**62. Do you feel trapped in your role as a caregiver?**	○	○	○	○
**63. Does caregiving cause difficulties in your relationship with your friends?**	○	○	○	○
**64. Do you find caregiving worthwhile?**	○	○	○	○
**65. Do you have a good relationship with the person you care for?**	○	○	○	○
**66. Do you feel that anyone appreciates you as a caregiver?**	○	○	○	○
**67. Do you feel you cope well as a caregiver?**	○	○	○	○
**68. Do you feel well supported by health and social services?**	○	○	○	○
**69. Overall, do you feel well supported in your role of caregiver?**	○	○	○	○

Information resources and need

In the past you might have looked for information in relation to your caregiving tasks. The following questions are about how you dealt with the search of information and how satisfied were you with the results of your search.

70. Have you ever looked for information in relation to your caregiving tasks from any source?

○ Yes

○ No to Q90

71. From the list below, please indicate 5 topics you have needed information on the most in relation to your caregiving tasks.

Check up to five topics

- ☐ Medical issues in relation to the person with SCI (e.g., bladder, bowel, skin, pain)
- ☐ Fitness/health promotion/nutrition in relation to the person with SCI
- ☐ SCI cure
- ☐ Equipment/assistive technology for the person with SCI
- ☐ Financial issues/health insurance/disability benefits in relation to the person with SCI
- ☐ Personal care assistants or providers for the person with SCI
- ☐ Home modifications
- ☐ Housing in relation to the person with SCI
- ☐ Legal issues/disability rights in relation to the person with SCI
- ☐ Sexuality/Fertility in relation to the person with SCI
- ☐ Employment in relation to the person with SCI
- ☐ Psychological issues (e.g., depression, anxiety) in relation to the person with SCI
- ☐ Social relations/maintaining relationships with others in relation to the person with SCI
- ☐ Other, please specify: ✐ ....

72. From the list below, please rank your top 3 current sources of information.

Check up to three options

- ☐ SCI specialist
- ☐ General physician
- ☐ Other healthcare professional
- ☐ SCI research organization/associations
- ☐ Support groups/Chats groups
- ☐ Others with SCI
- ☐ Family/Friends/Colleagues
- ☐ Magazines, journals or newsletters
- ☐ Books
- ☐ Television
- ☐ Brochure/pamphlets
- ☐ Internet web pages

73ff In general, how much would you trust information in relation to your caregiving tasks from each of the following?

	Not at all	A little	Some	A lot
73. Health professionals	○	○	○	○
74. Family/friends/coworkers	○	○	○	○
75. Support groups / Chats groups	○	○	○	○
76. Newspapers or magazines	○	○	○	○
77. Radio	○	○	○	○
78. Internet	○	○	○	○
79. Television	○	○	○	○
80. Government health agencies (e.g., Federal Office of Public Health)	○	○	○	○
81. Charitable organizations	○	○	○	○
82. Religious organizations and leaders	○	○	○	○

83ff Think about your most recent search for information in relation to your caregiving tasks. Based on the results of this search, how much do you agree or disagree with each of the following statements?

	Strongly agree	Some-what agree	Some-what disagree	Strongly disagree
83. It took a lot of effort to get the information I needed	○	○	○	○
84. I felt frustrated during my search for the information	○	○	○	○
85. I were concerned about the quality of the information	○	○	○	○
86. The information I found was hard to understand	○	○	○	○
87. I had difficulty making sense of information from multiple sources	○	○	○	○
88. I felt overwhelmed by the amount of information available	○	○	○	○

89. Overall, how confident are you that you could get advice or information in relation to your caregiving tasks if you needed it?

○ Completely confident
○ Very confident
○ Somewhat confident
○ A little confident
○ Not confident at all

Service Utilization

In this section, we would like to learn about the support you receive as a caregiver, additionally to professional care services the person with SCI may receive. What is your opinion on the usefulness of various services? Do they meet up your needs?

90. Who is the first contact person/institute when the person with SCI has a health problem?

- ○ General practitioner
- ○ Specialized doctor working in a private practice
- ○ Professional home care
- ○ Counselling institution such as ParaHelp
- ○ General hospital
- ○ Specialized clinic (SPZ Nottwil; Rehab Basel; Sion; Balgrist)
- ○ Other, please specify: ✎……………

91. In the past 12 months, which service have you utilized and how many times?

I have utilized service from professionals, namely

- ☐ Daycare in caring homes
  ✎……………. (number of times in the last 12 months)
- ☐ Respite care service during the day
  ✎……………. (number of times in the last 12 months)
- ☐ Respite care service during the night
  ✎……………. (number of times in the last 12 months)
- ☐ Home visit or social companionship
  ✎……………. (number of times in the last 12 months)
- ☐ Short term home care service/holiday respite
  ✎……………. (number of times in the last 12 months)
- ☐ Security or emergency calls of the SRK
  ✎……………. (number of times in the last 12 months)
- ☐ Advising service for yourself
  ✎……………. (number of times in the last 12 months)
- ☐ Training
  ✎……………. (number of times in the last 12 months)
- ☐ Support groups
  ✎……………. (number of times in the last 12 months)

○ Never utilized any service from professional service provider

92. If you did not utilize any service from professional providers listed in question 90 in the past 12 months, what were the reasons?

Check all that apply

- ☐ Never needed any service
- ☐ Not available in my region
- ☐ Costs too much
- ☐ I have sufficient support from family members or friends
- ☐ Had bad experience with service before
- ☐ Do not trust professional service providers

- ☐ The person who I take care does not feel comfortable with someone else
- ☐ Not satisfied with schedule arrangement/ the schedule is not flexible
- ☐ Other reasons: ✎……………………………………………………………

93. In reference to professional home care service, do you want to receive more service?

- ○ No, I am satisfied
- ○ Yes, I want to receive more service

When yes, please indicate the reason what keep you away from hiring more service.

Check all that apply

- ☐ Costs too much
- ☐ Had or heard of bad experience with service before
- ☐ Never trust professional service providers
- ☐ Feel strange to have someone stay in the house, who is not the family
- ☐ Not satisfied with their schedule arrangement / professional caregivers have inflexible schedule
- ☐ They do not provide the service that we need
- ☐ Other: ✎……………………………………………………………

94. Please indicate the reasons why you did not opt for permanent stays in nursing homes?

Check all that apply

- ☐ SCI person prefers to stay at home
- ☐ Difficult for relatives and friends to come visit SCI person
- ☐ Difficult to establish a social life in the nursing home
- ☐ Had or heard of bad experience with nursing home before
- ☐ Limits freedom and independency of the person with SCI
- ☐ Costs too much
- ☐ Other: ✎……………………………………………………………

95. Which of the following compensation schemes supports you for taking care of the person with SCI in the last 12 months?

Check all that apply

- ☐ Assistence allowance
- ☐ Caregiving credit
- ☐ I am employed through local Spitex as caregiver (with special qualification)
- ☐ Caregiving allowance ✎……………………………………………………………
- ☐ Andere, nämlich: ✎……………………………………………………………
- ○ None

Interpersonal relationships

The following questions focus on the amount of practical and emotional support that you receive from other persons.

96. Is there anyone, among the people who are close to you, with whom you can really talk about serious personal problems any time?

○ Yes, several persons
○ Yes, one person
○ No
○ Don't know

97ff To what extent can the following persons be available in case of need and show understanding, by talking with you for example, if 0 means "not at all" and 10 "a great deal"?

Even if you do not need any help should consider possible ways in which you could get support.

N/A means the person does not exist.

	Not at all									A great deal	N/A
	0	1	2	3	4	5	6	7	8	9	10	
**97. Your partner**	○	○	○	○	○	○	○	○	○	○	○	○
**98. Your children**	○	○	○	○	○	○	○	○	○	○	○	○
**99. Your mother**	○	○	○	○	○	○	○	○	○	○	○	○
**100. Your father**	○	○	○	○	○	○	○	○	○	○	○	○
**101. Your brothers and sisters**	○	○	○	○	○	○	○	○	○	○	○	○
**102. Your closes friends**	○	○	○	○	○	○	○	○	○	○	○	○
**103. Your colleagues**	○	○	○	○	○	○	○	○	○	○	○	○
**104. Other caregivers, peers**	○	○	○	○	○	○	○	○	○	○	○	○
**105. Your neighbors, other relatives**	○	○	○	○	○	○	○	○	○	○	○	○

106. Do you miss someone, with whom you can talk about serious personal problems any time?

○ Yes
○ No
○ Don't know

107ff If necessary, in your opinion, to what extent can the following persons provide you with practical help, this means concrete help or useful advice, if 0 means "not at all" and 10 "a great deal"?

Even if you do not need any help should consider possible ways in which you could get support; practical help = for example doing the shopping for you when sick, taking you to the doctor or giving useful advice in case of problems or when looking for specific information.

	Not at all										A great deal	N/A
	0	1	2	3	4	5	6	7	8	9	10	
**107. Your partner**	○	○	○	○	○	○	○	○	○	○	○	○

**108. Your children**	○	○	○	○	○	○	○	○	○	○	○
**109. Your mother**	○	○	○	○	○	○	○	○	○	○	○
**110. Your father**	○	○	○	○	○	○	○	○	○	○	○
**111. Your brothers and sisters**	○	○	○	○	○	○	○	○	○	○	○
**112. Your close friends**	○	○	○	○	○	○	○	○	○	○	○
**113. Your colleagues**	○	○	○	○	○	○	○	○	○	○	○
**114. Other caregivers, peers**	○	○	○	○	○	○	○	○	○	○	○
**115. Your neighbors, acquaintances**	○	○	○	○	○	○	○	○	○	○	○

116. How satisfied are you with your personal, social and family relationships, if 0 means "not at all satisfied" and 10 "completely satisfied"?

	Not satisfied at all										Completely satisfied
	0	1	2	3	4	5	6	7	8	9	10
Satisfaction with personal relationships	○	○	○	○	○	○	○	○	○	○	○

Community life, leisure time, and holidays

In this section, we would like to learn about your participation in community life, the leisure time activities you can enjoy beside your caregiving.

117ff The following are a number of activities. How frequently do you practice them?

**Frequency** / **Activities**	Every day	at least once a week	at least once a month	less than once a month	never
**Leisure activities**					
**117. Meeting friends, acquaintances, colleagues**	○	○	○	○	○
**118. Going to sport events**	○	○	○	○	○
**119. Sport (gym, jogging, volleyball, basketball etc), Walking, hiking in the mountain or in the countryside**	○	○	○	○	○
**120. Artistic activities and handicraft (instrument, singing, painting, drawing, doing sculptures, taking artistic pictures, DIY, gardening)**	○	○	○	○	○
**121. Going to an cultural event or site (cinema, theatre, opera, classic concert or museum etc)**	○	○	○	○	○
**122. Read (Books, newspaper or free daily newpaper, magazines)**	○	○	○	○	○

123. Playing video games (on computer, PlayStation, TV, handy etc.)	○	○	○	○	○
**Participation in community life**					
124. Participation in a social or cultural organization or club	○	○	○	○	○
125. Participation in political party	○	○	○	○	○
126. Participation in religious or spiritual groups	○	○	○	○	○

127. How many times did you leave home and go on holiday at least one week the last 12 months (without the person whom you take care of)?

✎…………… (number of times)

128. Think of this ladder as representing where people stand in Switzerland

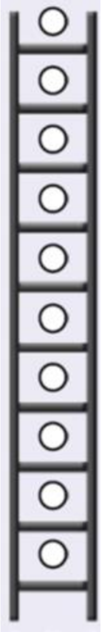

At the top of the ladder are the people who are the best off - those who have the most money, the most education and the most respected jobs. At the bottom are the people who are the worst off – who have the least money, least education, and the least respected jobs or no job. The higher up you are on this ladder, the closer you are to the people at the very top; the lower you are, the closer you are to the people at the very bottom.

Where would you place yourself on this ladder?

Please place a large X on the rung where you think you stand at this time in your life, relative to other people in Switzerland

The next few questions are about the person with spinal cord injury. We would like to learn about the situation of the person that you are taking care of.

Personal information

We would like to ask some questions about the person with spinal cord injury.

129. Please indicate his/her sex

○ Male

○ Female

130. Please indicate the year when the person was born.

✎…………… **please indicate the year (e.g.1976)**

131. Who lives in his/her household besides him/her?

Check all that apply

☐ Nobody, he/she lives alone

☐ Children under 14 years of age, number: ✎……………

☐ Persons between 14 and 64 years of age, number: ✎……………

- ☐ Persons over 64 years of age, number: ✎................

132. What is his/her current working situation?

Check all that apply

- ☐ Working ✎.............. % working quota
- ☐ Student (school, higher education, etc)
- ☐ Working for unpaid job (Re-training, unpaid internship, etc)
- ☐ Unemployed
- ☐ Housewife / househusband
- ☐ Retired due to the health condition
  - ○ Full retirement
  - ○ Part retirement
- ☐ Retired due to age
- ☐ Other, please specify: ✎...

133. Which financial support does he/she receive for obtaining caregiving?

Check all that apply

- ☐ Helplessness allowance
- ☐ Caregiving compensation
- ☐ Assistance allowance
- ☐ Complementary compensation
- ☐ Private insurance
- ☐ Others: ✎...............

134. If he/she does not live in the same household with you. What is the total household *gross* income of him/her on average per month?

Please take into account all persons living in his/her household.

- ○ Under CHF 1,500
- ○ CHF 1,500 to CHF 3,000
- ○ CHF 3,001 to CHF 4,500
- ○ CHF 4,501 to CHF 6,000
- ○ CHF 6,001 to CHF 7,500
- ○ CHF 7,501 to CHF 9,000
- ○ More than CHF 9,000

Lesion characteristics

In this section, we would like to learn about the spinal cord injury of the person.

135. Please indicate the reason of his/her spinal cord injury.

- ○ Because of an accident
- ○ Because of disease
- ○ Other reason, please specify ✎..............

136. Please describe the level of his/her spinal cord injury

- ○ Paraplegia
- ○ Tetraplegia

137. What was the characteristics of the injury?

- ○ Completely wheelchair dependent
- ○ Able to stand
- ○ Partly able to walk

138. Please indicate the year in which his/her spinal cord injury occurred

✐…………… (such as: 2011)

We thank you very much for participating in the survey!
